# Dynamic regulation of KIF15 phosphorylation and acetylation promotes focal adhesions disassembly in pancreatic cancer

**DOI:** 10.1038/s41419-022-05338-y

**Published:** 2022-10-25

**Authors:** Zhiwei He, Jie Wang, Jian Xu, Xueyi Jiang, Xinyuan Liu, Jianxin Jiang

**Affiliations:** Department of Hepatic-Biliary Surgery, Renmin Hospital of Wuhan University, 430060 Wuhan, China

**Keywords:** Oncogenes, Focal adhesion

## Abstract

Pancreatic cancer (PC) is prone to distant metastasis in the early stage, which is attributed to the strong migration ability of tumor cells. Focal adhesion turnover is essential for cancer cell metastasis, and the integrin recycling process is a key activation pathway for focal adhesion depolymerization. To identify the key motor protein involving in the integrin β1 recycling, we screened kinesin proteins involved in integrin β1 recycling using a kinesin family siRNA library and identified kinesin family 15 (KIF15) as a key regulator. KIF15 was upregulated in metastasis PC tissues and promoted PC cell migration and invasion. We identified KIF15 as a key component mediating integrin β1/FAK signaling that accelerated FA disassembly in a FAK-Y397-dependent manner. KIF15 recruited PI3K-C2α to promote integrin β1/FAK signaling and FA disassembly in a RAB11A-dependent manner. The C-terminal tail of KIF15 is required for the PI3K-C2α interaction and RAB11A activation. In addition, we also found that SIRT1-mediated acetylation of KIF15 is essential for KIF15 phosphorylation, which is the key activation event in motor protein function. Together, these findings indicate that KIF15 interacts with PI3K-C2α to promote FA turnover in PC cells by controlling the endosome recycling of integrin β1 in a SIRT1 acetylation modification-dependent manner, eventually promoting focal adhesions turnover and distant metastasis in PC.

## Introduction

Pancreatic cancer (PC) has an extremely higher mortality rate among all cancers [1]. PC patients with metastatic disease do not have the option of surgical resection and have a lower survival rate compared with patients with early detection [2]. Therefore, clarifying the biological and molecular mechanisms driving PC metastasis is important for the development of novel therapies for PC patients. Accumulating evidence has shown that focal adhesions (FAs) turnover is a critical event in local invasion and distant metastasis in the cancer cell, and phosphorylation and activation of focal adhesion kinase (FAK) at Y397 is necessary for FA turnover [3]. The main mechanism of FAK-mediated tumor cell tail FAs disassembly involves the following pathways [4–8]: phosphorylation of FAK recruits calpains to FAs, which degrade FAK and promote FA disassembly; FAK also regulates FA disassembly by activating RACK1, which in turn affects the activity of ERK located at FAs; and FAK interacts with the dynein Dyn2, affecting clathrin-dependent vesicle endocytosis to promote FAs disassembly. Studying the molecular mechanism underlying the phosphorylation and activation of FAK in tumor cells and the accelerated disintegration of FAs is of great significance for a better understanding of the increased invasion and metastasis observed in PC and the development of possible therapeutic molecular targets for PC.

A recent study revealed a different FAK-activation pathway: endosomes containing activated integrin are matured and sorted; while some become late endosomes and undergo lysosomal degradation, others become recycling endosomes that are transported to the ventral or tail of the cell membrane, the latter considered a novel pathway mediated FAK activation [9–11]. In the process of endosome recycling, integrin recruits FAK to the endosome and activates FAK through effector molecules such as PI(4,5)P2 and PI3K [12, 13]. Therefore, integrin internalization and recycling are important events that regulate FAK activation and FA disassembly. Current studies suggest that the internalization or recycling of integrins to the membrane depends on the directional transport of intracellular endosomes, and this process is regulated by Dyneins or Kinesins. Kinesin motor proteins function as a trailer for the directional transport of integrins in the integrin-mediated FA turnover in tumor and non-tumor cells [14–16]. Meanwhile, we previously found that KIF15 regulates PC cell growth and accelerating cell division by activating MAPK signaling [17]. The kinesin motor protein KIF15, also termed as kinesin-12, is involved in the regulation of internalization of α2 integrin, which participates in the transport of cell components, endosomes, and pathogens [18, 19]. In addition, phosphorylation of rat kinesin-12 at T1142 modulates rat cortical astrocytes migration, and phosphorylation of S1169 by Aurora A is required for targeting Kif15 to the spindle [20, 21]. Several studies showed that KIF15 is associated with cancer progression and tumorigenesis. Gao et al. reported that KIF15 shields the N-terminus of the androgen receptor and suppresses its ubiquitination-mediated degradation. KIF15 also functions as a scaffold to recruit USP14-mediated androgen receptor deubiquitination and upregulation, resulting in enzalutamide resistance and cell proliferation in prostate cancer [22]. Therefore, Investigation on KIF15-mediated integrins recycling is important to elucidate the molecular mechanisms of FAs turnover during PC metastasis.

Integrin β1 is an essential metastatic kinesin in pancreatic cancer, and its function in the activation of FAK and depolymerization of focal adhesions during its recycling to the membrane has been demonstrated [4, 23]. In this study, we screened kinesin motor proteins that were essential for integrin β1 recycling and FA turnover and identified KIF15 as a candidate factor. we demonstrated that KIF15 contributes to FAs turnover of PC through integrin b1 recycling and FAK activation. Importantly, the mutually exclusive mechanism of phosphorylation and acetylation modification of KIF15 is a key regulatory mechanism for its involvement in integrin b1 recycling trafficking.

## Materials and methods

### Cell culture

Human MIA PaCa-2 and PANC-1 cell lines were purchased from the American Type Culture Collection. The cells were grown in Dulbecco’s Modified Eagle’s Medium (DMEM, Gibco, USA). Cell cultures were supplemented with 10% fetal bovine serum (FBS, Gibco) and 0.5% penicillin and streptomycin (Gibco).

### Animal experiments

The protocol for the animal experiments was approved by the Renmin Hospital of Wuhan University Central Animal Laboratory. Six-week-old female BALB/c-nude mice were purchased from Weitonglihua (Beijing, China). Each nude mouse was injected via the tail vein with 2 × 10^6^ PANC-1 cells transfected with sh-Contol and shKIF15 lentivirus. After 8 weeks, the mice were sacrificed, and lung tissue sections were stained with hematoxylin–eosin for histological examination.

### Patient specimens

PC tissue specimens were obtained from the Department of Hepatic–Biliary–Pancreatic Surgery, Renmin Hospital of Wuhan University. The collection of specimens and clinical pathological data were approved by the Institutional Research Ethics Committee of Renmin Hospital of Wuhan University. The PC patients were diagnosed by clinical and histopathology diagnoses.

### siRNA library screening

A FlexiPlate siRNA library targeting 38 kinesin family genes in 96-well plates was obtained from Ribobio(Guangzhou, China). PC cells were seeded into plates (3000 cells/well) and cultured overnight. Cells were then transfected with siRNA in Lipofectamine 3000 at 5 nM for 48 h before erastin (10 μM) treatment for 24 h. The capture elisa detection was performed according to the integrin β1 recycling assays.

### Lentiviral vector production and transfection

Lentiviral vectors of KIF15, RAB11A, PI3K-C2α, and shRNAs of KIF15 were purchased from Genechem Company (Shanghai, China). Lentiviral vector transduction was performed as previously reported [17]. Lentiviral plasmids encoding shRNAs against control (shControl: 5′-UUCUCCGAACGUGUCACGUTT-3′) and KIF15 (shKIF15#1: 5′-GAAGTGAAGAGGCTCAAATT-3′, shKIF15#2: 5′-GGAACAAATGAGTGCTCTT-3′).

### Western blotting

Western blotting was performed as previously described [17]. Primary antibodies against KIF15, FAK, FAK-397, PXN, PXN-118, SRC, SRC-418, RAB11A, PI3K-C2α, and FLAG were purchased from Abcam. The Integrin Antibody Sampler Kit and antibody against GAPDH were purchased from Cell Signaling Technology. HRP-conjugated secondary antibodies, including polyclonal goat mouse IgG and polyclonal goat rabbit IgG, were purchased from Cell Signaling. GADPH was used as a normalization loading control. Expression levels were quantified by Imaging Systems (Thermo Fisher), and the relative expression values were quantified using Image J software.

### Immunoprecipitation and mass spectrometry analysis

Lysates from PC cells were incubated with primary antibody or IgG overnight at 4 °C. Affinity agarose beads were added to the lysates, and samples were shaken for 4 h, followed by washing of beads five times with pre-cooled IP wash buffer. The eluted proteins were boiled, denatured, and separated by gel electrophoresis, and gels were stained with Coomassie Blue. The band of interest was analyzed by mass spectrometry to identify the candidate proteins.

### Phos-Tag and western blot analysis

Phos-tag gel was made with 20 μM Phos-tag and used at a concentration of 20 µM in 8% SDS-acrylamide gels. Western blotting, lambda phosphatase treatment, and immunoprecipitation were performed as previously described [24]. Before being transferred to the nitrocellulose membrane, the gel of Mn2^+^-Phos-tag SDS-PAGE electrophoresis was softly rinsed with EDTA for 10 min. The following steps were consistent with conventional western blotting.

### Transwell assays

Transwell chambers were obtained from Corning for migration and invasion assays. Cells in serum-free DMEM (200 μl, 2 × 10^5^/ml) were placed in the upper chamber, and medium containing 10% FBS was placed in the bottom chamber. For invasion assays, Matrigel was coated in the upper chamber before cell seeding. The cells were then cultured for 24 h. Cells that did not pass through the upper chamber were wiped with a cotton swab, and the migrated or invaded cells were fixed with 4% paraformaldehyde for 30 min, stained with crystal violet, and counted.

### Immunofluorescence (IF) staining

Cells were seeded in confocal dishes to 30% confluence. The medium was aspirated, and the cells were washed twice with PBS and fixed with 4% paraformaldehyde for 30 min. After another wash with PBS, the cells were incubated in PBS containing 0.2% Triton X-100 for 10 min and then blocked in 5% BSA for 1 h. Primary antibodies for immunofluorescence were the same as those used for western blotting. The primary antibody or probe was added, and cells were incubated overnight at 4 °C. Cells were then incubated with fluorescent secondary antibody for 2 h. After washing three times with PBS, cells were sealed with glycerol and examined under a confocal microscope.

### Integrin recycling-mediated FA disassembly

This experiment was based on the previously reported β1 integrin endocytosis-mediated FA depolymerization model [25]. Each group of cells was cultured in a serum-free medium for 24 h and then cultured with nocodazole (10 μM) for 3 h. Integrin β1 antibody was added, and cells were incubated for 1 h; unbound antibody and nocodazole were washed away with pre-cooled PBS. Cell surface antibodies were removed by acid rinse, and cells were cultured at 37 °C or 4 °C for the indicated times (0 min, 30 min). Cells were fixed in paraformaldehyde, and after permeabilization, FAK or β1 integrin was detected by immunofluorescence assay. Staining was examined under a laser confocal microscope.

### Integrin internalization/recycling assay (capture ELISA)

Integrin internalization and recycling ELISA assays were performed as previously described (10). The cells were biotinylated with cell surface integrin using Sulfo-NHS-SS biotin (0.2 mg/ml) in cold PBS. The cells were then incubated at 37 °C for a specified time, and the control cells were kept on ice. Cell surface biotin was eluted with MesNa (50 mM) in PBS, followed by washing MesNa in the culture solution with iodoacetamide (20 mM) in PBS. Cells were then lysed and protein concentration was quantified. Biotinylated protein was immunoprecipitated from equal amounts of total protein using streptavidin agarose beads. After beads were washed with pre-cooled PBS, integrin α5 and β1 were detected by ELISA.

In the integrin recycling assay, biotin labeling was performed as described above. The cells were cultured at 37 °C for 30 min, and cell surface biotin was eluted with the above solution (MesNa, iodoacetamide). The cells were again placed at 37 °C and a second cell surface biotin elution was performed for the indicated times. Finally, the ELISA detection step in the internalization experiment was repeated to detect recycling integrin.

### Protein-degradation assays

PC cells in six-well plates were transfected with indicated lentivirus plasmid and incubated for 24 h. Cycloheximide at 10 μg/ml was added to cells for the indicated times. Cells were lysed in RIPA buffer, and a western blot was performed to determine integrin expression, using GAPDH for normalization. The untreated control was set to 100%, and integrin expression at each time point was calculated as the percentage of the untreated control.

### Statistical analysis

Results are presented as mean ± standard deviation (SD). Student’s *t* test was used to assess the differences between experimental and control groups. Pearson’s test was used to analyze the correlation between the two targets in different specimens. All experiments were performed three times.

## Results

### Screening for candidate Kinesin superfamilies (KIFs) involved in integrin β1 recycling

**Fig. 1:**
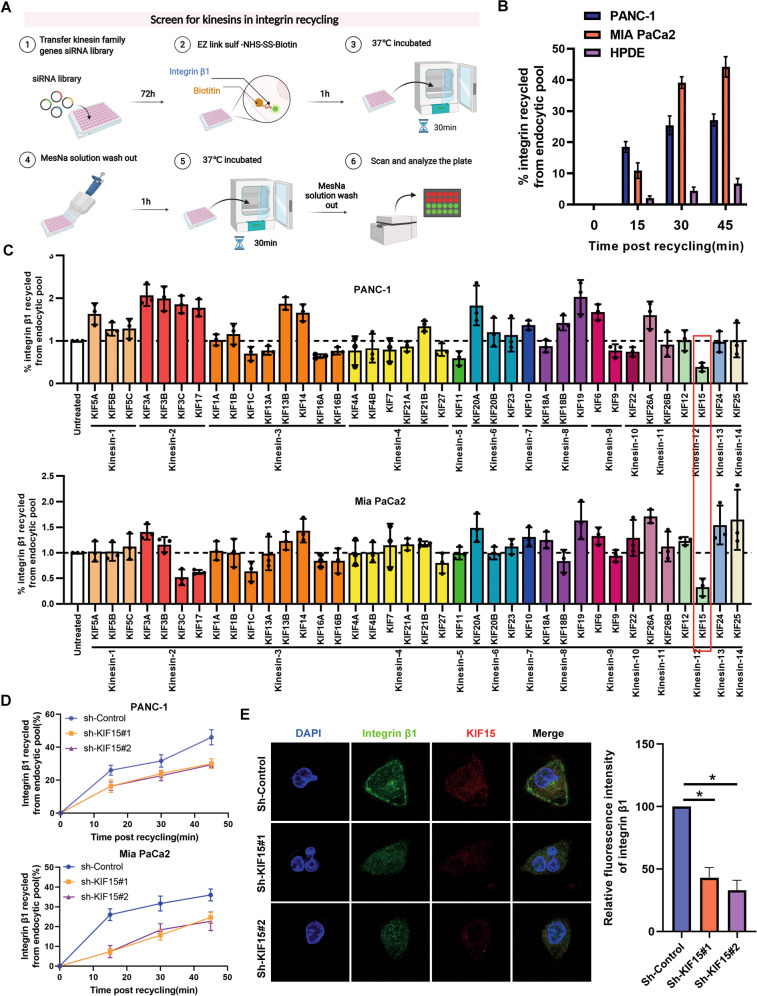
Screening for candidate Kinesin superfamilies (KIFs) involved in integrin β1 recycling. **A** The schematic diagram of integrin β1 recycling assay was used to screen integrin β1 recycling-regulating kinesins. The absorbance reflected the integrin β1 recycling content in an indicated cell transfected with different KIFs siRNAs. **B** integrin β1 recycling assay was used to evaluate the integrin β1 recycling content in indicated time. **C** PANC-1 and MIA PaCa-2 cells were seeded into a 96-well plate coated with 38 different Kinesin siRNAs, and then performed to detect the integrin β1 recycling content. The absorbance was detected at 45 min post-recycling. **D** integrin β1 recycling assay was used to validate the effect of KIF15 on integrin recycling. **E** integrin β1 cell membrane expression was determined by immunofluorescence. All data represent means ± SEM; ****P < 0.0001, ***P < 0.001, **P < 0.01; *P < 0.05; two-tailed Student’s *t* test.

To screen KIFs involved in integrin β1 recycling trafficking, we constructed a capture-ELISA model to the high-content analysis of the integrin β1 recycling regulation (Fig. 1A). PC cells were seeded in 96-well plates pre-loaded with a KIF siRNA library. capture ELISA was performed for integrin exocytosis, and integrin β1 recycling content was analyzed on the microplate reader. In control cells, the amount of recycled integrin β1 was time-dependent, with the highest level at 45 min (Fig. 1B). According to the screening assay, the KIF15 silenced sample showed the lowest amount of recycled integrin β1 (Fig. 1C). We next validated the effect of KIF15 in integrin β1 recycling, and capture ELISA and immunofluorescence showed that integrin β1 was remarkably decreased in the KIF15 knockdown groups compared with shControl group (Fig. 1D, E). Notably, KIF15 does not affect integrin endocytosis in PC cells (Supplementary Fig. S1). These results demonstrated that KIF15 is a vital kinesin motor protein in integrin β1 recycling.

### KIF15 correlated with PC metastasis and accelerated FA disassembly

**Fig. 2:**
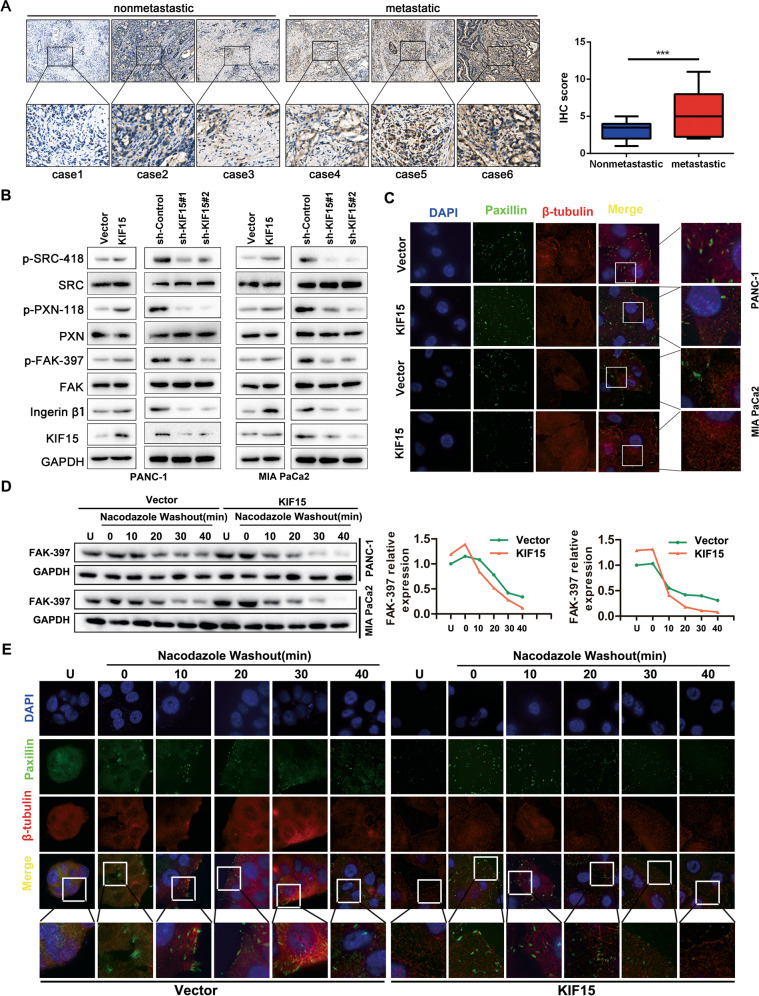
KIF15 correlated with PC metastasis and accelerated FA disassembly. **A** Immunohistochemical analysis of KIF15 expression in non-metastatic and metastatic pancreatic cancer tissues. **B** Immunofluorescence analysis of focal adhesions (Paxillin: green, microtubules: red) in the tails of cells of negative control (Vector) and KIF15-overexpressed PC cells. **C** Western blot analysis of the Integrin/FAK signaling pathway in cells with interference or overexpression of KIF15. **D** PANC-1 and Mia PaCa-2 cells were incubated with 10 mM nacodazole, followed by drug washout for 40 min. Relative levels of FAK phosphorylated on Y397 were quantified by scanning densitometry and normalized to levels of GAPDH. **E** PANC-1 cell was incubated with 10 mM nacodazole, followed by drug washout for 40 min. Representative immunofluorescence analysis of focal adhesion disassembly (Paxillin: green, microtubules: red). All data represent means ± SEM; ***P < 0.001, **P < 0.01; *P < 0.05; two-tailed Student’s *t* test.

To examine the relation between KIF15 expression and PC distant metastasis, we performed immunohistochemistry on PC patient specimens and found that KIF15 was overexpressed in PC tissues with distant metastasis compared with non-metastasis specimens (Fig. 2A). KIF15 knockdown significantly inhibited the migration and invasion ability of PC cells in in vitro and in vivo assays (Supplementary Fig. S2A–C). Integrin β1 recycling was confirmed that promoted p-FAK-397-dependent focal adhesion disassembly and metastasis. We found that KIF15 overexpression increased integrin β1 expression and the phosphorylation of FAK, SRC and PXN, while KIF15 knockdown had the opposite effects (Fig. 2B). In wound healing assays, the area of FAs was decreased in the KIF15-overexpressed PC cells determined by Immunofluorescence (Fig. 2C). In the FAs disassembly cell model, KIF15 significantly promoted FAK dephosphorylation, which indicated FA turnover (Fig. 2D). We also observed the turnover of adhesion plaques in this cell model by confocal microscopy, and the rate of FAs of cells in the KIF15 overexpression group was significantly increased compared with Vector group (Fig. 2E and Supplementary Fig. S2D). Together, these results demonstrate that KIF15 promotes PC metastasis and accelerated FA disassembly.

### Integrin β1 recycling is essential for KIF15 promoting FA turnover and metastasis in PC cells

**Fig. 3:**
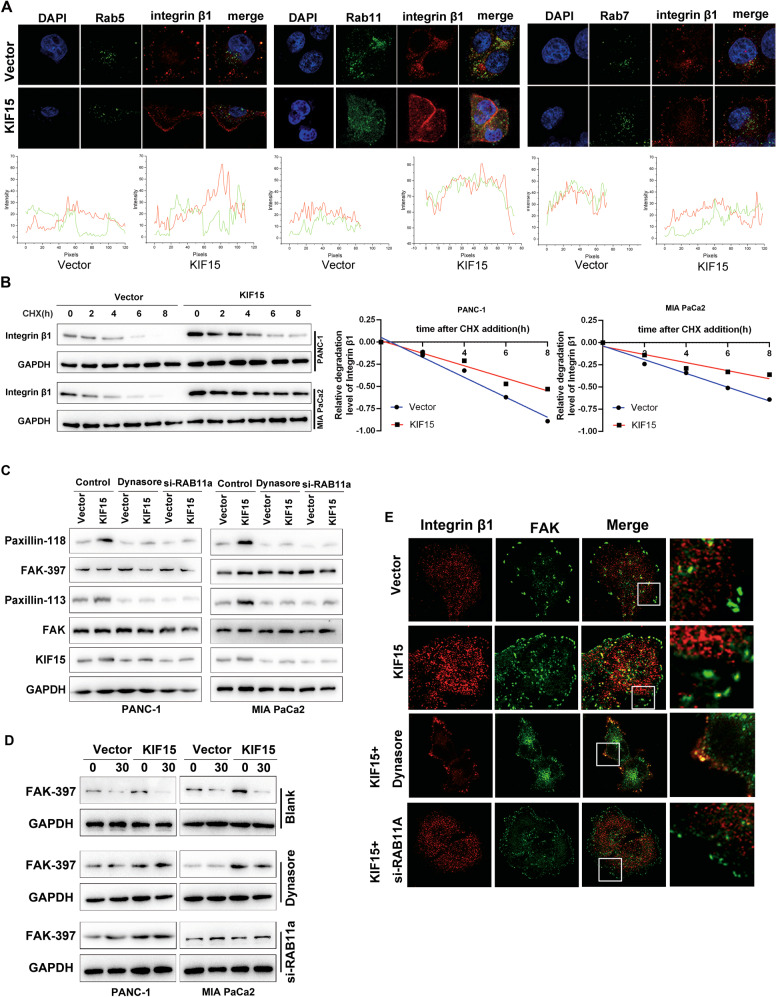
Integrin β1 recycling is essential for KIF15 promoting FA turnover and metastasis in PC cells. **A** The co-localization of Integrin β1 with early endosome (Rab5), recycling endosome (Rab11), and late endosome (Rab7) markers was analyzed by immunofluorescence. **B** Cycloheximide (CHX, 50 μg/ml) was used to treat Vector, KIF15-overexpressed PC cells for 0, 2, 4, 6, and 8 h. Integrin β1 expression was examined by western blotting. Relative levels of integrin β1 were quantified by scanning densitometry and normalized to the level of GAPDH. **C** Western blot analysis of FAK pathway activity in KIF15-overexpressed PC cells that treated with endosome trafficking inhibitor Dynasore or RAB11 siRNA. **D** Western blot analysis of p-FAK-397 after nacodazole washout in indicated cells. The KIF15-overexpressed PC Cells were treated with endosome trafficking inhibitor Dynasore or RAB11 siRNA. **E** Immunofluorescence analysis of the focal adhesions disassembly in indicated cells. All data represent means ± SEM; ***P < 0.001, **P < 0.01; *P < 0.05; two-tailed Student’s *t* test.

To clarify whether integrin β1 recycling is essential for KIF15 promoting PC cell FA turnover, a model of integrin recycling with adhesion disassembly was used to monitor the involvement of integrin β1 in PANC-1 cells. Integrin β1 was initially distributed in the cytoplasm within many small FAs dots around the membrane in control and KIF15-overexpressing PANC-1 cells. After eluting nocodazole under 37 °C, the FAs of the KIF15-overexpressing cells disassembled faster compared with the Control group, while the turnover rate of FAs of the control and overexpression groups eluted under 4 °C was almost the same (Supplementary Fig. S3A). The endosome trafficking inhibitor Dynasore and RAB11A siRNA were used to block integrin β1 recycling (Supplementary Fig. S3B). In addition, KIF15-overexpressed induction of migration and invasion ability was partly inhibited by inhibiting integrin β1 recycling (Supplementary Fig. S3C). To clarify the mechanism of integrin β1 recycling and upregulation by KIF15 overexpression, we performed an immunofluorescence assay to determine the location of integrin β1 with early/recycling/late endosome, their marker protein is RAB5, RAB11, RAB7, respectively. The results indicated that KIF15 promoted the co-localization of integrin β1 and RAB11A, inhibited the co-localization of integrin β1 and RAB7 and there was no significant change in the co-localization between integrin and RAB5 (Fig. 3A). Moreover, protein-degradation experiments suggest that KIF15 can significantly inhibit the degradation of integrin β1 (Fig. 3B). Furthermore, the KIF15-dependent FAK activation and FA turnover were inhibited by blocking integrin β1 recycling (Fig. 3C–E). These results show that integrin β1 recycling is essential for KIF15 promoting the depolymerization of FAs in PC cell metastasis.

### The KIF15 and PI3K-C2α interaction is essential for integrin β1 recycling on RAB11A-activated endosomes

**Fig. 4:**
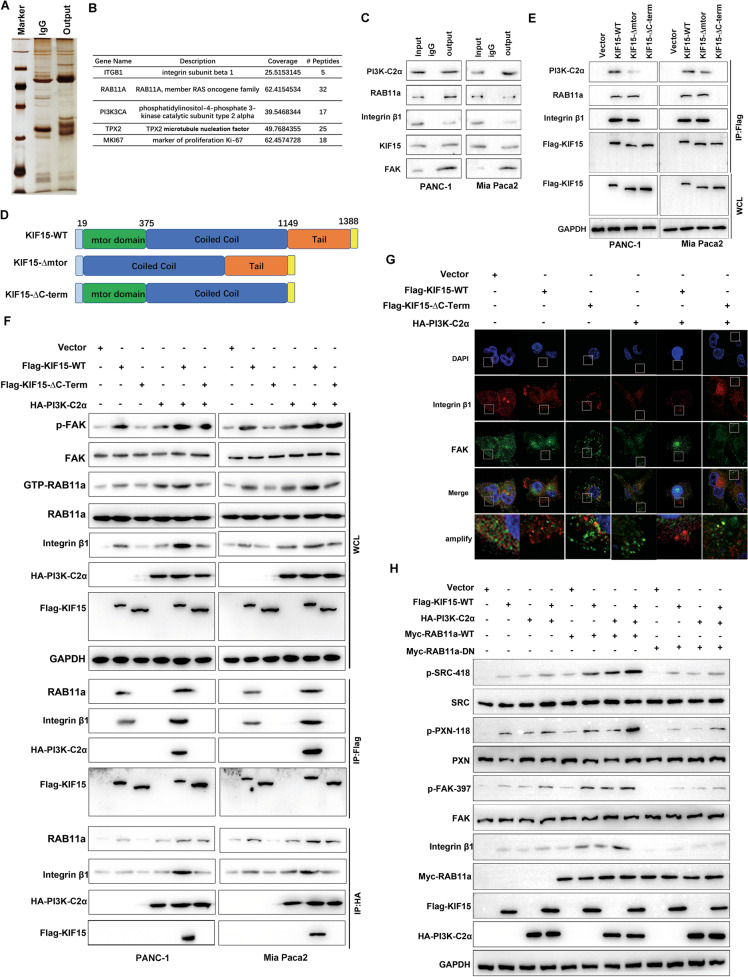
The KIF15 and PI3K-C2α interaction is essential for integrin recycling on RAB11A-activated endosomes. **A**, **B** Proteins that interacted with KIF15 were identified by silver staining and mass spectrometry. **C** Co-immunoprecipitation analysis of the interaction between KIF15 and Rab11, PI3K-C2α, RAB11A, integrin β1. **D** Schematic model of the domain structure of wild-type KIF15 (KIF15-WT) and KIF15 mutants depleting either aa19-375 (KIF15-Δmtor) or aa1149-1388(KIF15-ΔC-term). **E** Wild-type KIF15 and different mutation constructs plasmid were transfected into PC cells, anti-Flag antibody was used for the co-immunoprecipitation, and substrate proteins were detected using western blot. **F** The effect of KIF15 or KIF15-ΔC-term interacted with PI3K-C2α on the integrin β1/FAK pathway activity in whole cell lysis by western blot and the interaction of KIF15, Rab11A Integrin β1, PI3K-C2α by Flag or HA immunoprecipitation assay. **G** Immunofluorescence analysis of the interaction of KIF15-WT or KIF15-ΔC-term with PI3K-C2α on the depolymerization of focal adhesions in PANC-1 cell. **H** Western blot analysis of the effects of KIF15 and PI3K-C2α complexes on integrin signaling in the wild-type or inactive state of RAB11A. All data represent means ± SEM; ***P < 0.001, **P < 0.01; *P < 0.05; two-tailed Student’s *t* test.

First, we used immunoprecipitation with mass spectrometry analysis to determine endosome trafficking effector proteins that interact with KIF15. We found PI3K-C2α probably was the target exerted function involving in endosome recycling interacted with KIF15, and TPX2, KI-67 were reported previously (Fig. 4A, B). Immunoprecipitation confirmed that KIF15 interacted with PI3K-C2α, RAB11A, and integrin β1 (Fig. 4C). As the C-terminal domain is highly conserved in KIF15, we constructed truncated mutants to detect the interacting domain (Fig. 4D and Supplementary Fig. S4A). KIF15 lacking the C-terminal domain lost PI3K-C2α, RAB11A, and integrin β1 binding function (Fig. 4E). KIF15 lacking the C-terminal domain also showed reduced integrin β1/FAK signaling pathway activation. Moreover, after overexpressed PI3K-C2α, the effect of KIF15 promoting integrin β1/FAK was more remarkably, and the interaction of KIF15, PI3K-C2α, RAB11A, and integrin β1 also increased, while there was no effect in KIF15 lacking the C-terminal domain group (Fig. 4F). Immunofluorescence demonstrated that FA turnover was significantly promoted by KIF15, co-expression of PI3K-C2α strengthened this effect, but had no effect on KIF15 without the C-terminal domain (Fig. 4G). Furthermore, the complex of KIF15 and PI3K-C2α can significantly activate the FAK signaling pathway co-transfected with RAB11A, but its activation effect is significantly inhibited in the state of RAB11A inactivate (Fig. 4H). Meanwhile, integrin β1 recycling and the metastasis ability were partly inhibited in KIF15 lacking the C-terminal domain or transfected with si-PI3K-C2α, compared to KIF15-overexpressed PC cells (Supplementary Fig. S4B–D). Therefore, the interaction of PI3K-C2α with the KIF15 C-terminal domain is necessary for integrin β1 recycling on RAB11A-activated endosomes.

### Phosphorylation and acetylation of KIF15 is opposite to PI3K-C2α interaction and FA depolymerization

**Fig. 5:**
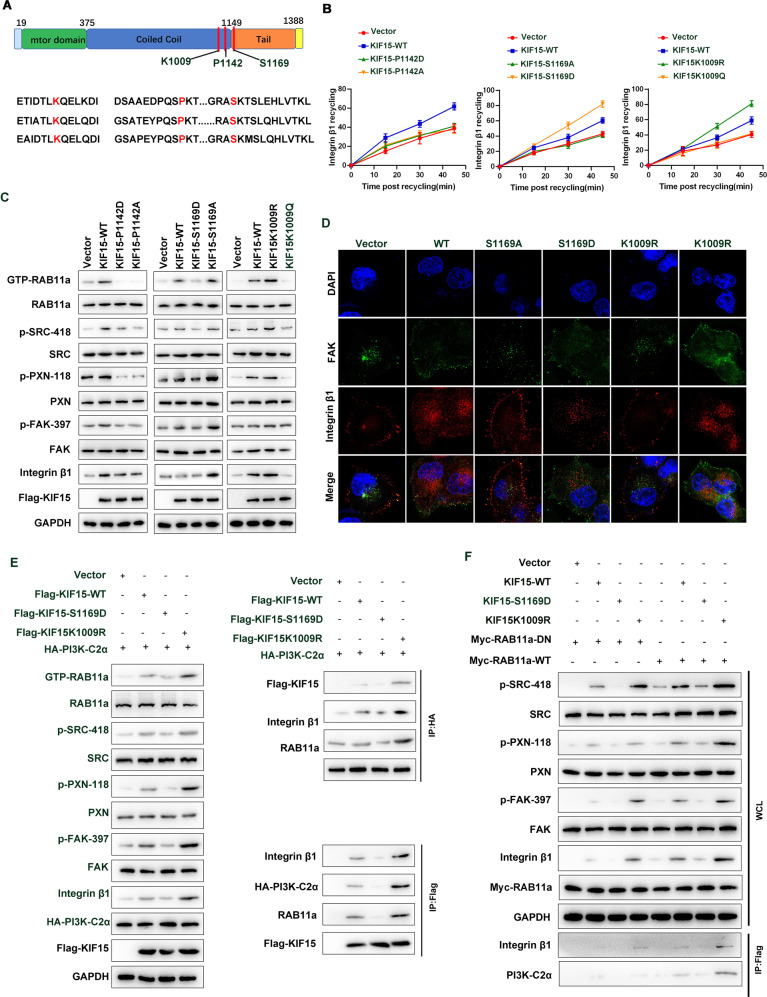
Phosphorylation and acetylation of KIF15 is opposite to PI3K-C2α interaction and FA depolymerization. **A** Schematic model demonstrated the conservation analysis of the acetylation and phosphorylation of KIF15 in various species. **B** Capture-Elisa assay analysis of the integrin β1 recycling content in acetylation of K1009 inactivating or activating mutant of KIF15, S1169 and P1142 inactivating and activating mutant. **C** The effect of acetylation of K1009 inactivating or activating mutant of KIF15, S1169, and P1142 inactivating and activating mutant on the integrin β1/FAK pathway activity in whole cell lysis by western blot. **D** Immunofluorescence analysis of the acetylation of K1009 inactivating or activating mutant of KIF15, S1169 inactivating and activating mutant on the depolymerization of focal adhesions in PANC-1 cell. **E** The effect of K1009 activating mutant of KIF15, S1169 activating mutant on the integrin β1/FAK pathway activity in whole cell lysis by western blot and the interaction of KIF15, Rab11A Integrin β1, PI3K-C2α by Flag or HA immunoprecipitation assay. **F** Western blot analysis of the effects of K1009 deacetylation mutant of KIF15, S1169 activating mutant on integrin β1/FAK signaling in the wild-type or inactive state of RAB11A. All data represent means ± SEM; ***P < 0.001, **P < 0.01; *P < 0.05; two-tailed Student’s *t* test.

The C-terminal domain of KIF15 was reported to interact with cargo or endosomes, and some post-translation modifications may regulate trafficking and protein binding. function. Based on the UNIPROT database (www.uniprot.org), we found that KIF15 has three modification sites near the C-terminus, we constructed K1009, S1169, P1142 constitutively active and inactive mutants (Fig. 5A). Capture ELISA indicated that P1142 phosphorylation has no biological function, while S1169 phosphorylation or K1009 deacetylation could promote the integrin β1 recycling (Fig. 5B). In addition, we also found S1169 phosphorylation or K1009 deacetylation promoted integrin β1/FAK and FA depolymerization and FAK signaling pathway (Fig. 5C, D). The interaction between KIF15 and integrin β1, PI3K-C2α, and RAB11A is also weakened and strengthened by S1169 phosphorylation and K1009 deacetylation (Fig. 5E). KIF15 dephosphorylation and deacetylation resulted in increased FAK signaling and RAB11A activity, respectively, but this effect was weakened in RAB11A inactivated mutation (Fig. 5F). The above results indicated that KIF15 phosphorylation and deacetylation can promote RAB11A-dependent integrin β1 recycling and FAK signaling pathway activation, respectively.

### SIRT1 deacetylated KIF15 mediating integrin β1 recycling and FAK signaling activation

**Fig. 6:**
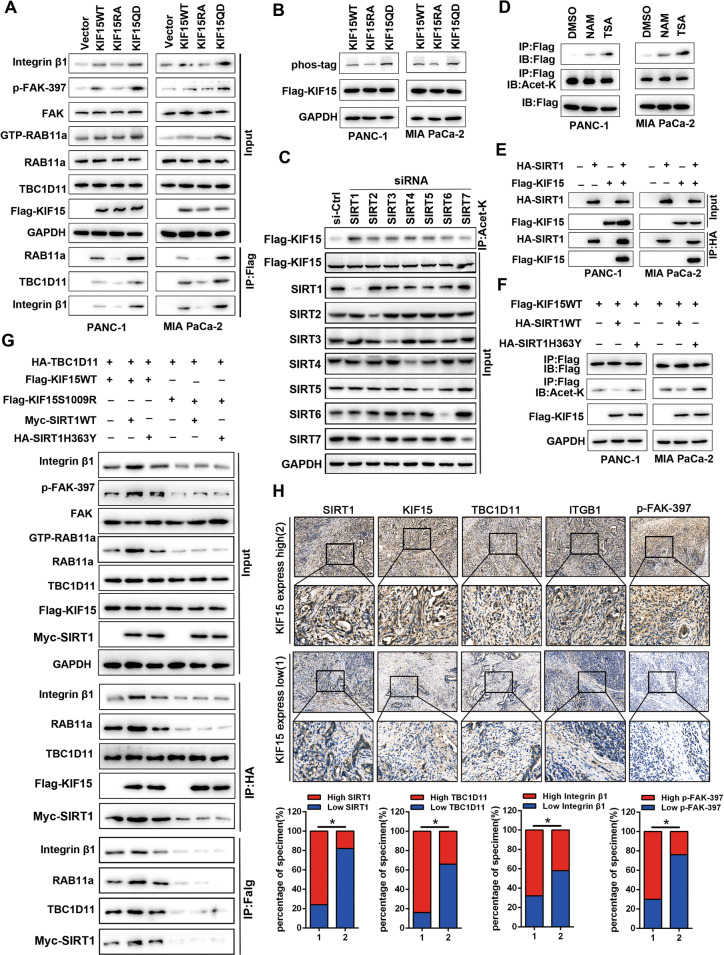
SIRT1 deacetylated KIF15 mediating integrin β1 recycling and FAK signaling activation. **A** The effect of simultaneously acetylated and phosphorylated (K1009Q + S1169D, KIF15-RA) or simultaneously deacetylated and dephosphorylated (K1009Q + S1169A, KIF15-QD) on the integrin β1/FAK pathway activity in whole cell lysis by western blot. **B** Phos-Tag and western blot analysis of the phosphorylation KIF15 expression level in transfected with acetylation of K1009 inactivating or activating mutant PC cells. **C** Western blot analysis of the effects of acetylation inhibitors on KIF15 acetylation levels. **D** Western blot analysis of the effects of different SIRTs siRNA on KIF15 acetylation levels. **E** Co-immunoprecipitation analysis of the interaction between KIF15 and SIRT1. **F** Western blot analysis of the effects of SIRT1-WT or SIRT1-H363Y inactive mutation on KIF15 acetylation levels. **G** The effect of KIF15-WT or K1009 activating mutant of KIF15 on the integrin β1/FAK pathway activity in the wild-type or inactive state of SIRT1 by western blot and the interaction of KIF15, Rab11A, Integrin β1, PI3K-C2α by Flag or HA immunoprecipitation assay. **H** Immunohistochemical analysis of the correlation of KIF15 and RAB11A, Integrin β1, PI3K-C2α, SIRT1 in pancreatic cancer tissues. All data represent means ± SEM; ***P < 0.001, **P < 0.01; *P < 0.05; two-tailed Student’s *t* test.

KIF15 is simultaneously acetylated and phosphorylated (K1009Q + S1169D, KIF15-QD) or simultaneously deacetylated and dephosphorylated (K1009R + S1169A, KIF15-RA). The results suggested that the biological function of KIF15-RA was lost, while the biological function of KIF15-QD remained unchanged in the integrin β1/FAK activation (Fig. 6A). In addition, the migration and invasion ability and integrin β1 recycling were promoted in KIF15-QD group PC cell, compared to KIF15-WT PC cells (Supplementary Fig. S6A, B). Therefore, phosphorylation of KIF15 is a key modification that leads to the activation of the integrin β1/FAK signaling pathway. However, we found that the level of KIF15 phosphorylation was regulated by KIF15 acetylation (Fig. 6B). Meanwhile, the KIF15 acetylation level was significantly upregulated in PC treated with SIRTs and HDACs inhibitors (TSA and NAM) (Supplementary Fig. S6C), but the integrin β1/FAK pathway was inhibited (Supplementary Fig. S6E). Therefore, we examined upstream deacetylases of KIF15, as this may be a critical protein that regulates the function of KIF15. We used SIRTs and HDACs inhibitors to acetylate KIF15, and the results showed that Sirtuins inhibitors was more effective (Fig. 6C). We next knocked down each SIRT family member and examined KIF15 acetylation level. The results indicated that SIRT1 may be a deacetylase for KIF15 (Fig. 6D). Co-immunoprecipitation experiments confirmed the interaction between KIF15 and SIRT1 (Fig. 6E). When SIRT1 deacetylation activation is inactivated, its deacetylation effect on KIF15 almost disappears (Fig. 6F). SIRT1 mediating KIF15 deacetylation significantly enhanced the biological function of RAB11A, integrin β1/FAK activation and the interaction of KIF15, RAB11A, integrin β1, and PI3K-2α, while the SIRT1 deacetylation inactivation mutant has no such effect (Fig. 6G). In addition, KIF15 was positively correlated with SIRT1, RAB11A, integrin β1, and PI3K-2α (Fig. 6H). These data demonstrated that SIRT1 promoted deacetylated KIF15-mediated integrin β1 recycling and FAK signaling pathway activation.

### The SIRT1 inhibitor EX527 exhibits broad synergy with the KIF15 inhibitor KIF15-IN in KIF15-overexpressing PC cell lines

**Fig. 7:**
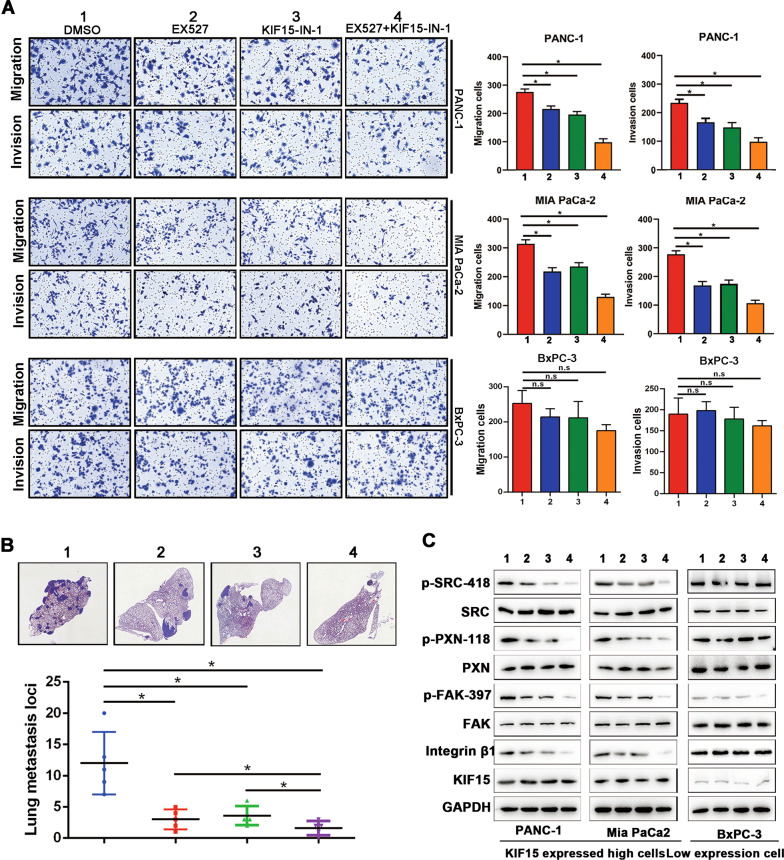
The SIRT1 inhibitor EX527 exhibits broad synergy with the KIF15 inhibitor KIF15-IN in KIF15-overexpressing PC cell lines. **A** Transwell migration and invasion assays showed that treatment with either SIRT1 inhibitor EX527 or KIF15 inhibitor KIF15-IN significantly inhibited the proliferation of KIF15-overexpressed PANC-1 and Mia PaCa-2 cells, and the combination of the two inhibitors had the most obvious inhibitory effect, but not KIF15-deficient BxPC-3 cell. **B** In vivo assessment of the effect of either SIRT1 inhibitor EX527, KIF15 inhibitor KIF15-IN, and the combination of the two inhibitors on invasion and migration of PC cells by nude mouse lung metastasis assay. **C** Western blot analysis indicated a significant increase in the integrin β1/FAK signaling pathway after being treated with either SIRT1 inhibitor EX527, KIF15 inhibitor KIF15-IN and the combination of the two inhibitors in KIF15-overexpressed PANC-1 or Mia PaCa-2 cells and KIF15-deficient BxPC-3 cell.

EX527 is a highly selective deacetylase SIRT1 inhibitor, and KIF15-IN is a highly selective inhibitor of KIF15, both of them are considered to have a significant inhibitory effect on tumor proliferation. To investigate the effects of drugs on cell invasion and metastasis, we selected drug concentrations with an inhibition rate of 90% for drug combination (Supplementary Fig. S7A–C). After EX527 and KIF15-IN-1 were added to cells, the viability of the three PC lines was higher than 80%, indicating these concentrations had little effect on the viability of PC cells (Supplementary Fig. S7D). Transwell experiments showed that the two inhibitors have a significant inhibitory effect on the invasion and metastasis ability of PANC-1 and MIA PaCa-2 cells highly expressing KIF15, with no effect in BxPC-3 cell with low KIF15 expression (Fig. 7A). The combination of EX527 and KIF15-IN-1 significantly inhibited metastasis ability of PC cells in vivo and integrin β1/FAK signaling pathway (Fig. 7B, C). Meanwhile, HE staining of mouse heart, kidney, stomach, and liver indicated that the above drugs had almost no toxicity in mice (Supplementary Fig. S7E). Therefore, we conclude that due to the acetylation modification of SIRT1 on KIF15, the integrin β1/FAK pathway of KIF15-overexpressed pancreatic cancer cell was activated, providing a prerequisite for the metastasis inhibition effect of EX527 combined with KIF15-IN-1.

## Discussion

Invasion and migration are essential steps in metastasis and are important reasons for the poor prognosis of PC patients. However, the mechanisms underlying these processes are only partially understood. In this regard, the role of FA disassembly and endosome trafficking in metastasis is emerging as a crucial issue in cancer research [26]. Accumulating evidence shows that KIF15 is involved in cell motility, integrin trafficking and cytoskeleton rearrangement of both normal and tumor cells [20, 27, 28]. We previously showed that KIF15 activates the MAPK signaling pathway to promote the proliferation of PC cells [17]. These reports indicate that KIF15 is involved in tumor progression and metastasis.

The mechanism by which KIF15 promotes cell migration has remained elusive. Here, we observed that in KIF15 is significantly upregulated in tumors of patients with lymph node metastasis and distant metastasis. in vitro and in vivo metastasis experiments showed that KIF15 overexpression significantly promotes cell motility. Analyzing microarray mRNA expression data for KIF15 revealed that KIF15 is significantly associated with cell adhesion. Adhesion of tumor cells is closely related to FAK signaling and integrin molecules [29]. Our study showed that KIF15 is required for activation of FAK signaling and integrin β1 endosome recycling, which accelerates FA disassembly. However, the molecular mechanism by which KIF15 promotes integrin recycling-mediated FAK activation and FA disassembly is currently unclear. The above results were first proposed that FAK phosphorylation-induced FA disassembly occurs through KIF15-mediated endosome recycling of integrin β1.

FAs is associated with FAK phosphorylation, but the FAK-activation mechanism in Kinesins-induced FA disassembly is unclear [28]. However, research has shown that the endosome trafficking of integrins is required for the activation of FAK and FA disassembly, and this mechanism is considered a new FAK-activation pathway [30]. These endosome trafficking association with FA disassembly at the tail of cell meets the previous results that cells treated with endosome trafficking blockers showed faster in FAs turnover [31]. In the above molecular mechanism of FAK activation and FA disassembly, KIF15 may provide motor-mediated transport of integrin β1 on microtubules, recruiting and activating FAK in the process. Alanko et al. showed that integrin not only binds and activates FAK at FAs, but it also recruits and activates FAK during the transport of endosomes back to the membrane [12]. KIF15 functions as a motor protein for endosome transport by interacting with microtubules [32]. Thus, through the ability of KIF15 to regulate transport of endosomes to the membrane, microtubules may act as a molecular motor to deliver cargo (integrin β1) to accelerate FA disassembly. Therefore, our results imply that KIF15 promotes FA disassembly by accelerating integrin β1 endosome trafficking.

We further explored the mechanism of KIF15 promotion of FA disassembly by accelerating endosome trafficking to enhance cancer cell migration. Immunoprecipitation combined with mass spectrometry revealed that KIF15 binds to RAB11A and PI3K-C2α. Previous studies showed that KIF15 promotes the dissolution of FAs, which is mediated by recycling endosomes. RAB11A-dependent recycling of endosomes was demonstrated to be an essential molecular mechanism for integrin pathway activation [33]. PI3K-C2α stabilizes the mitotic spindle and promotes cell division in breast cancer [34]. PI3K-C2α is a RAB11A-binding protein participating in RAB11A activation on endosomes, and PI3K-C2α-derived phosphatidylinositol 3-phosphate (PtdIns3P) is necessary to control the localization and activity of RAB11A. However, while PI3K-C2α co-localized with RAB11A, they did not co-immunoprecipitate with each other, implying that RAB11A may indirectly interact with PI3K-C2α [35]. Hence, we speculate that KIF15 might act as a scaffold to recruit RAB11A and PI3K-C2α and coordinate RAB11A activity and localization. Notably, we found that KIF15 interacts with RAB11A and PI3K-C2α via the C-terminus region of KIF15, with molecular and cellular effects. However, KIF15 does not affect the expression level of RAB11A. The Rabs family of proteins are essential vesicle transport effectors that mediate the abnormal activation of endosome signaling pathways depending on the form of GTP binding [36, 37]. Previous studies showed that the C-terminal domain of KIF15 is a key functional domain that binds to and regulates vesicles. Therefore, the differences in FA disassembly in cells expressing wild-type KIF15 or KIF15 C may be from the interaction between KIF15 and RAB11A. In addition, the C-terminal domain is likely a key functional domain of KIF15-mediated RAB11A activation. On the basis of previous studies [38], we speculate that KIF15 may bind GEFs and RAB11A for GDP/GTP conversion, which is KIF15 C-terminal domain-dependent. The C-terminus of KIF15 contains phosphorylation sites and acetylation sites. Emerging research has indicated that deacetylases modulate cell function by regulating the acetylation and phosphorylation level of key proteins [39, 40]. In our study, phosphorylated KIF15 at S1169 not T1142 site significantly recruited more PI3K-C2α around RAB11A, causing RAB11A activation. Furthermore, deacetylation of KIF15 at K1009 site by SIRT1 showed a consistent effect on the activation of RAB11A. We then mutated acetylation or phosphorylation sites and further confirmed that STRT1-mediated deacetylation at K1009 site promoted KIF15 phosphorylation at S1169 site and recruited more PI3K-C2α around RAB11A and activated RAB11A. RAB11A-controlled endosome trafficking of integrin β1 promoted FAK activation and FA disassembly.

**Fig. 8:**
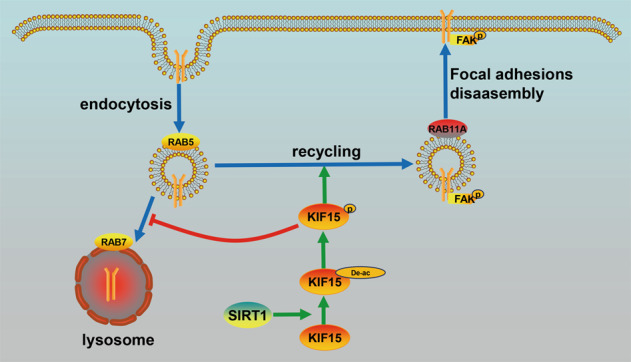
Schematic diagram to show dynamic regulation of KIF15 phosphorylation and acetylation promoting focal adhesions disassembly in pancreatic cancer.

Our results indicated that the effect of KIF15 on integrin β1 endosome recycling is of great significance in understanding FAK activation and FAs disassembly (Fig. 8). Our findings suggest that KIF15 potentially interacted with PI3K-C2α mediated RAB11A activation to facilitate β1 endosome trafficking and FAK activation. The C-terminal domain of KIF15 is required to interact with PI3K-C2α dependent on SIRT1-mediated deacetylation at K1009 and/or phosphorylation at S1169. Activated KIF15 recruited PI3K-C2α around RAB11A and activated RAB11A, leading to endosome trafficking of integrin β1 back to the plasma membrane and activating FAK-mediated FA disassembly. The exploration of KIF15-mediated endosome trafficking of integrin β1 will help uncover the related pancreatic cancer distant metastasis mechanisms.

## Supplementary information

Supplementary Data

aj-checklist.pdf

Original Data File

## Data Availability

The datasets supporting the conclusions of this article are included within the article and its additional files.

## References

[1] Siegel RL, Miller KD, Fuchs HE, Jemal A (2021). Cancer statistics, 2021. CA Cancer J Clin.

[2] Long J, Luo GP, Xiao ZW, Liu ZQ, Guo M, Liu L (2014). Cancer statistics: current diagnosis and treatment of pancreatic cancer in Shanghai, China. Cancer Lett.

[3] Horton ER, Byron A, Askari JA, Ng DHJ, Millon-Fremillon A, Robertson J (2015). Definition of a consensus integrin adhesome and its dynamics during adhesion complex assembly and disassembly. Nat Cell Biol.

[4] Nader GP, Ezratty EJ, Gundersen GGFAK (2016). talin and PIPKIgamma regulate endocytosed integrin activation to polarize focal adhesion assembly. Nat Cell Biol.

[5] Zaidel-Bar R, Ballestrem C, Kam Z, Geiger B (2003). Early molecular events in the assembly of matrix adhesions at the leading edge of migrating cells. J Cell Sci.

[6] Kerstein PC, Patel KM, Gomez TM (2017). Calpain-mediated proteolysis of talin and FAK regulates adhesion dynamics necessary for axon guidance. J Neurosci.

[7] Klimova Z, Braborec V, Maninova M, Caslavsky J, Weber MJ, Vomastek T (2016). Symmetry breaking in spreading RAT2 fibroblasts requires the MAPK/ERK pathway scaffold RACK1 that integrates FAK, p190A-RhoGAP and ERK2 signaling. Biochim Biophys Acta.

[8] Wong BS, Shea DJ, Mistriotis P, Tuntithavornwat S, Law RA, Bieber JM (2019). A direct podocalyxin-dynamin-2 interaction regulates cytoskeletal dynamics to promote migration and metastasis in pancreatic cancer cells. Cancer Res.

[9] Cullen PJ (2008). Endosomal sorting and signalling: an emerging role for sorting nexins. Nat Rev Mol Cell Biol.

[10] Dozynkiewicz MA, Jamieson NB, Macpherson I, Grindlay J, van den Berghe PV, von Thun A (2012). Rab25 and CLIC3 collaborate to promote integrin recycling from late endosomes/lysosomes and drive cancer progression. Dev Cell.

[11] Grant BD, Donaldson JG (2009). Pathways and mechanisms of endocytic recycling. Nat Rev Mol Cell Biol.

[12] Alanko J, Mai A, Jacquemet G, Schauer K, Kaukonen R, Saari M (2015). Integrin endosomal signalling suppresses anoikis. Nat Cell Biol.

[13] Alanko J, Ivaska J (2016). Endosomes: emerging platforms for integrin-mediated FAK signalling. Trends Cell Biol.

[14] Ahmed SM, Theriault BL, Uppalapati M, Chiu CW, Gallie BL, Sidhu SS (2012). KIF14 negatively regulates Rap1a-Radil signaling during breast cancer progression. J Cell Biol.

[15] Siddiqui N, Zwetsloot AJ, Bachmann A, Roth D, Hussain H, Brandt J (2019). PTPN21 and Hook3 relieve KIF1C autoinhibition and activate intracellular transport. Nat Commun.

[16] Uchiyama Y, Sakaguchi M, Terabayashi T, Inenaga T, Inoue S, Kobayashi C (2010). Kif26b, a kinesin family gene, regulates adhesion of the embryonic kidney mesenchyme. Proc Natl Acad Sci USA.

[17] Wang J, Guo X, Xie C, Jiang J (2017). KIF15 promotes pancreatic cancer proliferation via the MEK-ERK signalling pathway. Br J Cancer.

[18] Eskova A, Knapp B, Matelska D, Reusing S, Arjonen A, Lisauskas T (2014). An RNAi screen identifies KIF15 as a novel regulator of the endocytic trafficking of integrin. J Cell Sci.

[19] Hirokawa N, Noda Y, Tanaka Y, Niwa S (2009). Kinesin superfamily motor proteins and intracellular transport. Nat Rev Mol Cell Biol.

[20] Feng J, Hu Z, Chen H, Hua J, Wu R, Dong Z (2016). Depletion of kinesin-12, a myosin-IIB-interacting protein, promotes migration of cortical astrocytes. J Cell Sci.

[21] van Heesbeen R, Raaijmakers JA, Tanenbaum ME, Halim VA, Lelieveld D, Lieftink C (2017). Aurora A, MCAK, and Kif18b promote Eg5-independent spindle formation. Chromosoma.

[22] Gao L, Zhang W, Zhang J, Liu J, Sun F, Liu H (2021). KIF15-mediated stabilization of AR and AR-V7 contributes to enzalutamide resistance in prostate cancer. Cancer Res.

[23] Huang X, Ye Q, Chen M, Li A, Mi W, Fang Y (2019). N-glycosylation-defective splice variants of neuropilin-1 promote metastasis by activating endosomal signals. Nat Commun.

[24] Huo FC, Pan YJ, Li TT, Mou J, Pei DS (2019). PAK5 promotes the migration and invasion of cervical cancer cells by phosphorylating SATB1. Cell Death Differ.

[25] Steinberg F, Heesom KJ, Bass MD, Cullen PJ (2012). SNX17 protects integrins from degradation by sorting between lysosomal and recycling pathways. J Cell Biol.

[26] Mendoza P, Ortiz R, Diaz J, Quest AF, Leyton L, Stupack D (2013). Rab5 activation promotes focal adhesion disassembly, migration and invasiveness in tumor cells. J Cell Sci.

[27] Buster DW, Baird DH, Yu W, Solowska JM, Chauviere M, Mazurek A (2003). Expression of the mitotic kinesin Kif15 in postmitotic neurons: implications for neuronal migration and development. J Neurocytol.

[28] Ezratty EJ, Partridge MA, Gundersen GG (2005). Microtubule-induced focal adhesion disassembly is mediated by dynamin and focal adhesion kinase. Nat Cell Biol.

[29] Spiess M, Hernandez-Varas P, Oddone A, Olofsson H, Blom H, Waithe D (2018). Active and inactive beta1 integrins segregate into distinct nanoclusters in focal adhesions. J Cell Biol.

[30] Stehbens SJ, Paszek M, Pemble H, Ettinger A, Gierke S, Wittmann T (2014). CLASPs link focal-adhesion-associated microtubule capture to localized exocytosis and adhesion site turnover. Nat Cell Biol.

[31] Villari G, Jayo A, Zanet J, Fitch B, Serrels B, Frame M (2015). A direct interaction between fascin and microtubules contributes to adhesion dynamics and cell migration. J Cell Sci.

[32] Drechsler H, McAinsh AD (2016). Kinesin-12 motors cooperate to suppress microtubule catastrophes and drive the formation of parallel microtubule bundles. Proc Natl Acad Sci USA.

[33] Hulsbusch N, Solis GP, Katanaev VL, Stuermer CA (2015). Reggie-1/Flotillin-2 regulates integrin trafficking and focal adhesion turnover via Rab11a. Eur J Cell Biol.

[34] Gulluni F, Martini M, De Santis MC, Campa CC, Ghigo A, Margaria JP (2017). Mitotic spindle assembly and genomic stability in breast cancer require PI3K-C2alpha scaffolding function. Cancer Cell.

[35] Campa CC, Margaria JP, Derle A, Del Giudice M, De Santis MC, Gozzelino L (2018). Rab11 activity and PtdIns(3)P turnover removes recycling cargo from endosomes. Nat Chem Biol.

[36] Langemeyer L, Frohlich F, Ungermann C (2018). Rab GTPase function in endosome and lysosome biogenesis. Trends Cell Biol.

[37] Stenmark H (2009). Rab GTPases as coordinators of vesicle traffic. Nat Rev Mol Cell Biol.

[38] Novick P (2016). Regulation of membrane traffic by Rab GEF and GAP cascades. Small GTPases.

[39] Yang P, Xu C, Reece EA, Chen X, Zhong J, Zhan M (2019). Tip60- and sirtuin 2-regulated MARCKS acetylation and phosphorylation are required for diabetic embryopathy. Nat Commun.

[40] Zhang W, Feng Y, Guo Q, Guo W, Xu H, Li X (2020). SIRT1 modulates cell cycle progression by regulating CHK2 acetylation-phosphorylation. Cell Death Differ.

