# Supplementary material for: Dynamic regulation of KIF15 phosphorylation and acetylation promotes focal adhesions disassembly in pancreatic cancer
Source: Cell Death Dis. 2022 Oct 25;13(10):896. doi: 10.1038/s41419-022-05338-y (PMC9592618; doi:10.1038/s41419-022-05338-y)

**Figure S1, related to Figure 1.**

(A) Capture Elisa assay was used to validated the effect of KIF15 on integrin β1 endocytosis
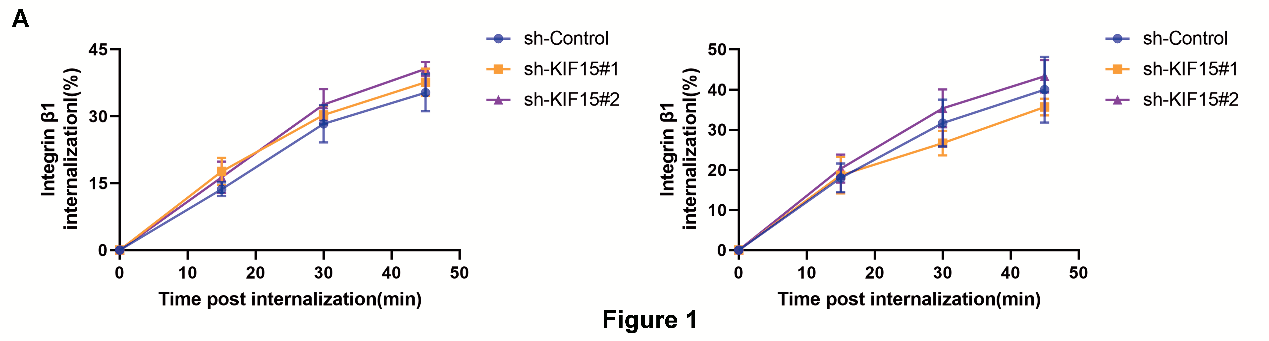


**MIA PaCa2**

**PANC-1**

**Figure S2, related to Figure 2**

(A-B) Transwell assay was used to evaluate the effect of KIF15 on the migration and invasion of PC cells. (C) In vivo assessment of the effect of KIF15 on invasion and migration of PC cells by nude mouse lung metastasis assay. (D) Mia PaCa2 cells was incubated with 10 mM nacodazole, followed by drug washout for 40 min. Representative immunofluorescence analysis of focal adhesion disassembly (Paxillin: green, microtubules: red).


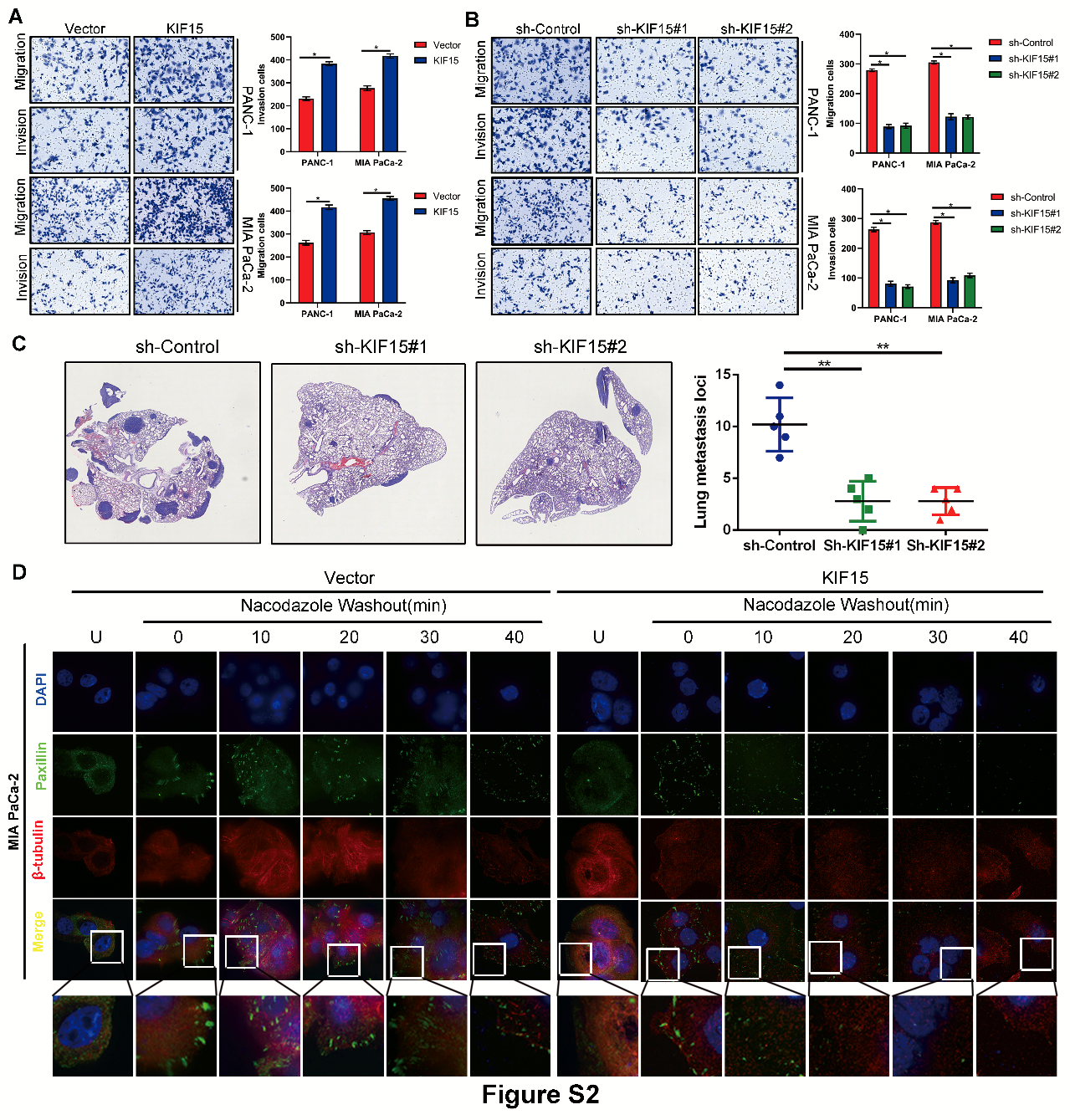


**Figure S3, related to Figure 3**

(A) Nacodazole-treated PC cells were incubated with integrin β1 antibody at 4°C to label cell surface β1 integrin. After removal of unbound antibodies and nacodazole, incubation of cells continued at 37°C or 4°C for the indicated times and cells were then immediately fixed and immunostained to visualize FAK (red in merged image) and integrin β1 (green in merged image). (B) Capture Elisa assay analysis of the Integrin β1 recycling content in indicated cells. The KIF15 overexpressed PC Cells were treated with endosome trafficking inhibitor Dynasore or RAB11 siRNA. (C) Transwell assay was used to evaluate the effect of endosome trafficking inhibitor Dynasore or RAB11 siRNA on the migration and invasion of KIF15 overexpressed PC cells.


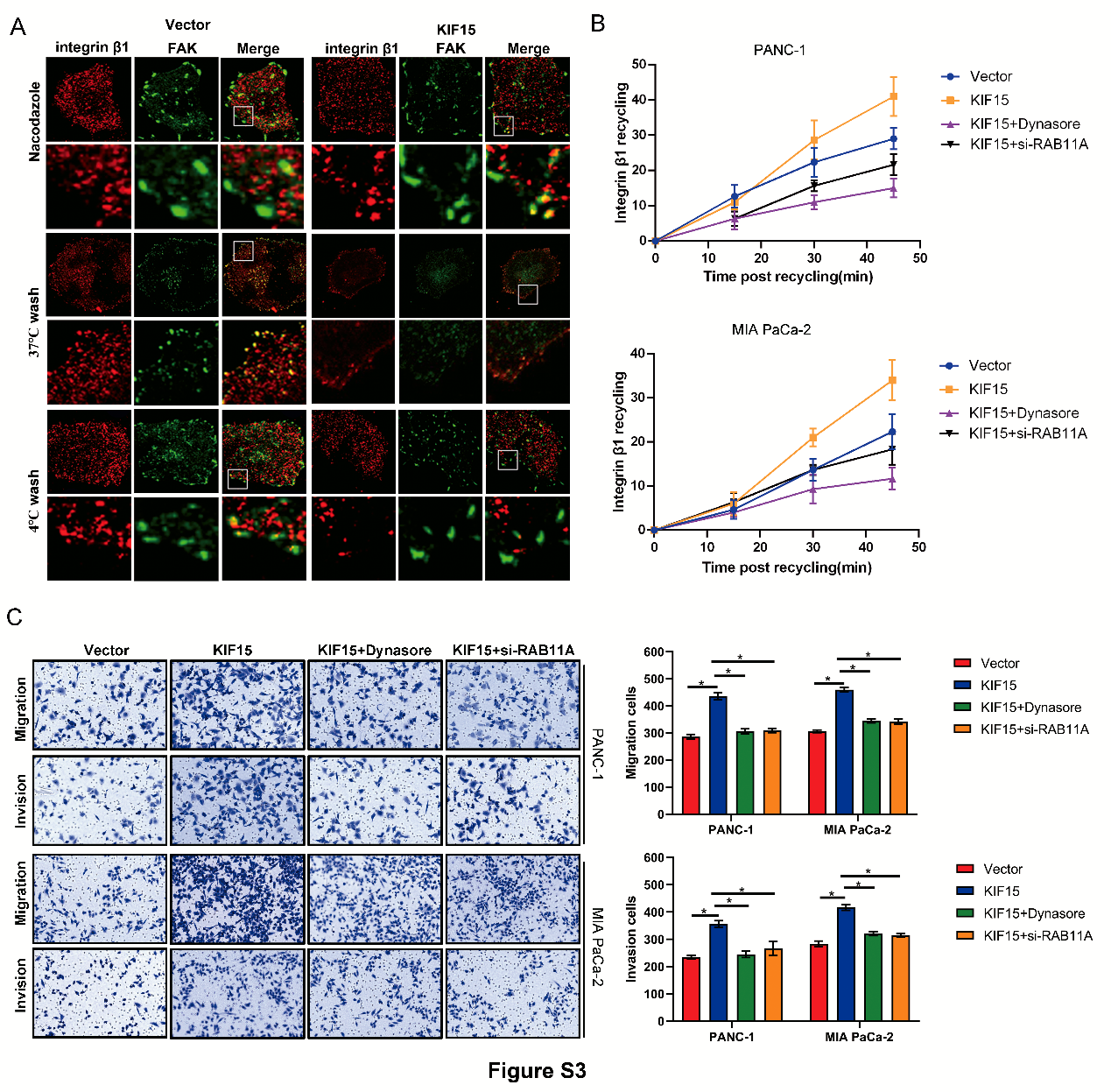


**Figure S4, related to Figure 4**

(A) Conservation analysis of the C-terminal tail domain of KIF15 in various species. (B) Transwell assay was used to evaluate the effect of KIF15-WT and KIF15-ΔC-term on the migration and invasion of PC cells. (C) Transwell assay was used to evaluate the effect of KIF15 overexpressed and transfected with PI3K-C2α siRNA on the migration and invasion of PC cells. (D) Capture Elisa assay analysis of KIF15-WT or KIF15-ΔC-term with PI3K-C2α on the depolymerization of integrin β1 recycling in PC cells.


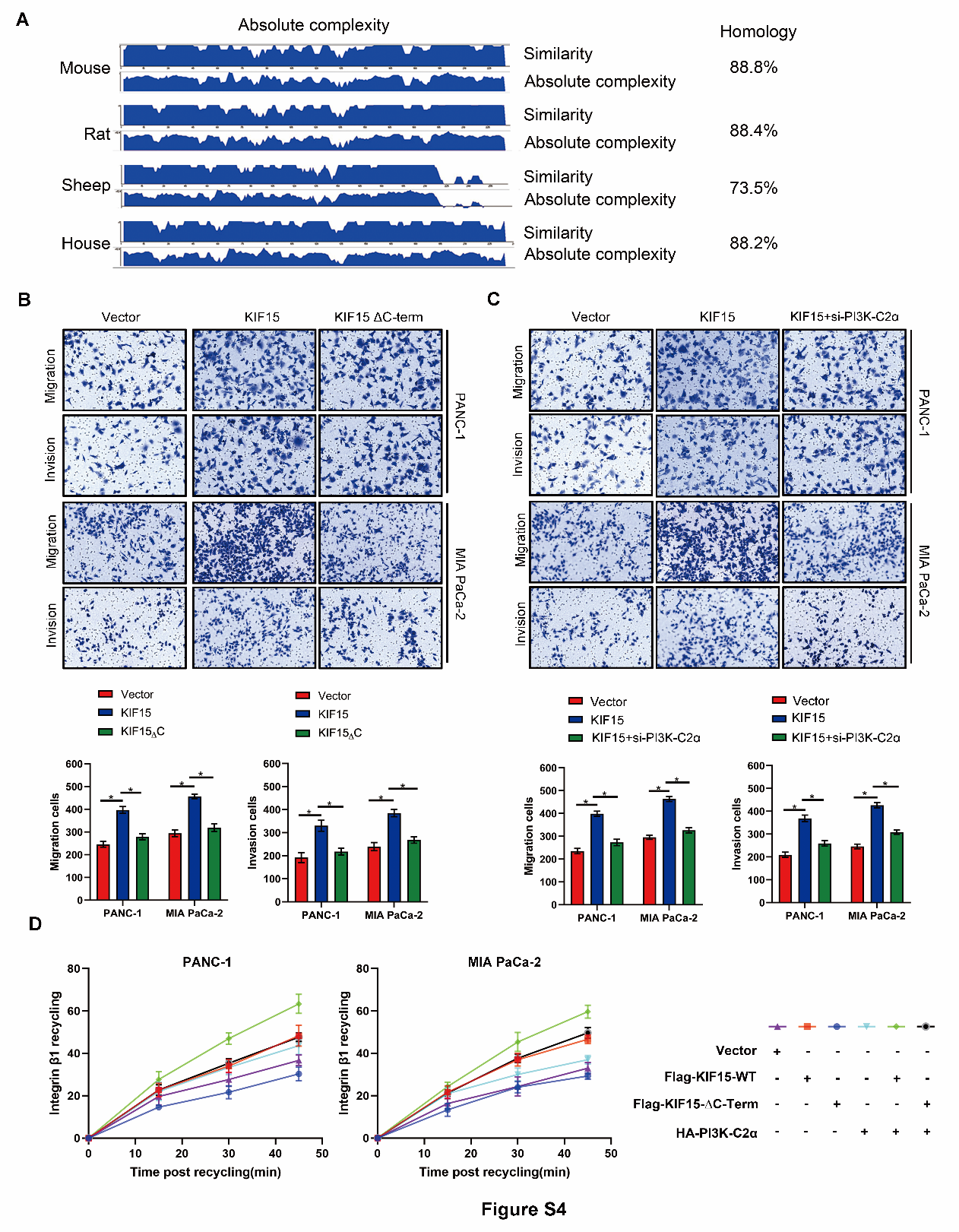


**Figure S5, related to Figure 5**

(A) Transwell assay was used to evaluate the effect of K1009 activating mutant of KIF15, S1169 activating mutant on the migration and invasion of PC cells.


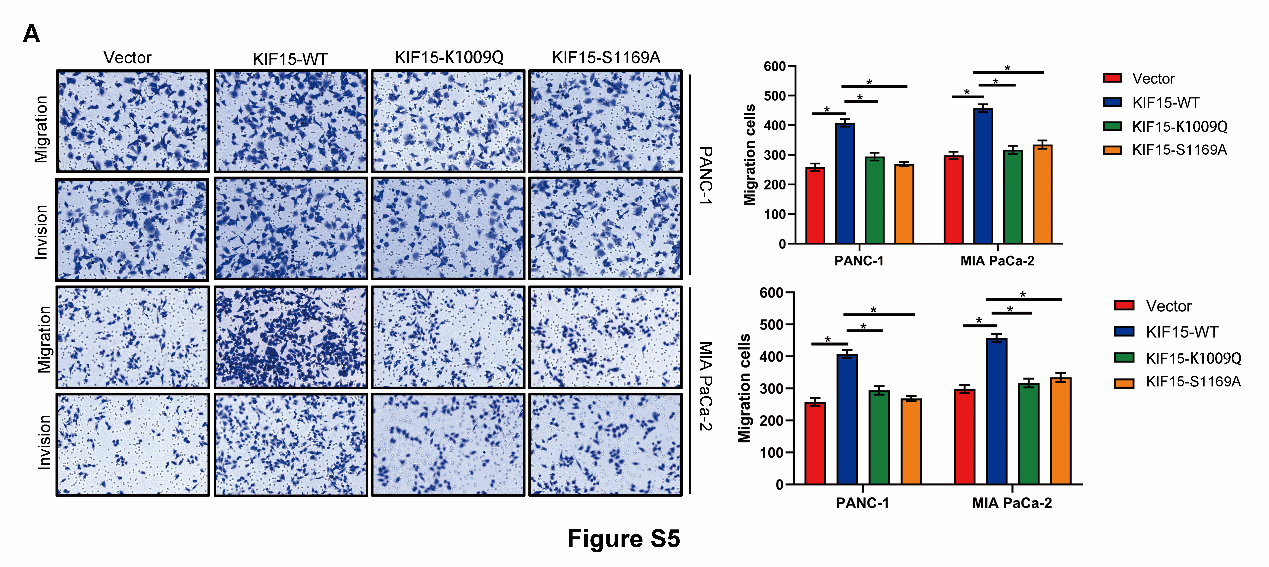


**Figure S6, related to Figure 6**

(A) The effect of simultaneously acetylated and phosphorylated (K1009Q+S1169D，KIF15-QD) or simultaneously deacetylated and dephosphorylated (K1009R+S1169A，KIF15-RA) on the PC migration and invasion ability. (B) The effect of KIF15-RD and KIF15-QA on the PC cell integrin β1 recycling. (C-D) KIF15 acetylation level in the PC treated with Deacetylation inhibitor TSA and NAM. (E) Westernblot assay was performed to detect integrin β1/FAK pathway activity and co-immunoprecipitation was used to validate the interaction of KIF15, RAB11A and PI3K-C2α in the PC treated with Deacetylation inhibitor TSA and NAM.


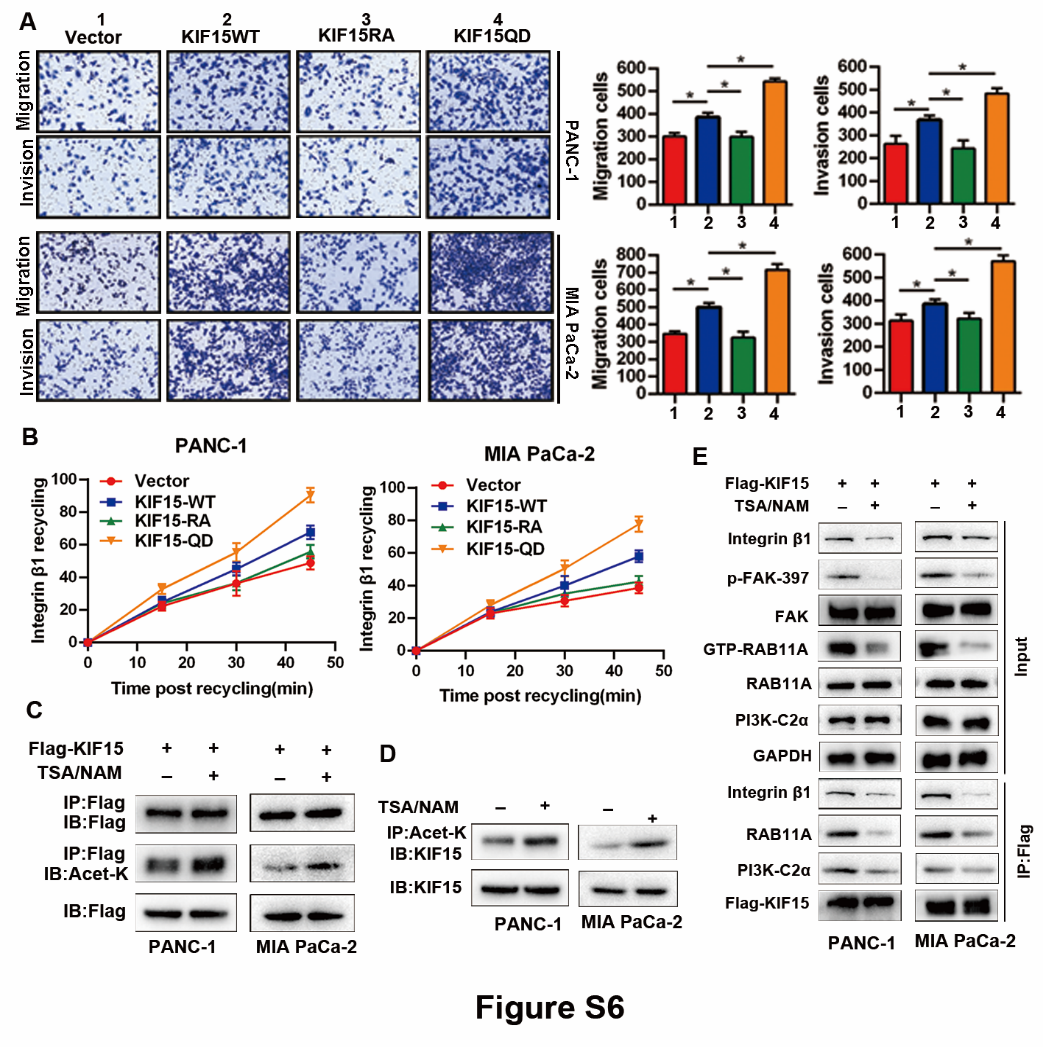


**Figure S7, related to Figure 7**

(A-C) Cell viability analysis of the 90% inhibition of EX 527 and KIF15-IN-1 in the PC cells. (D) Cell viability analysis of the combination of the two inhibitors. (E) HE staining to observe the morphological changes of mouse organs treated with either SIRT1 inhibitor EX527 or KIF15 inhibitor KIF15-IN and the combination of the two inhibitors.


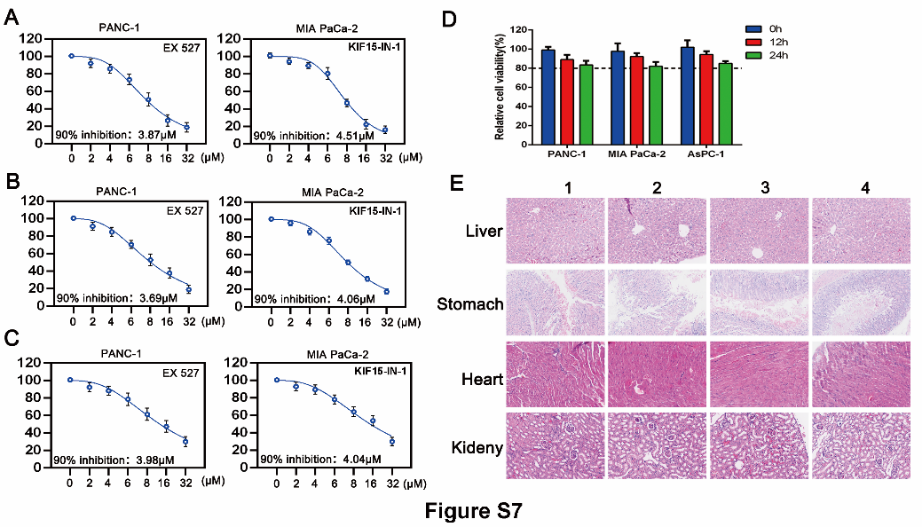

Supplement: Supplementary file 1 — Supplementary Data [file 41419_2022_5338_MOESM1_ESM.docx]
