# Supplementary material for: Dynamic regulation of KIF15 phosphorylation and acetylation promotes focal adhesions disassembly in pancreatic cancer
Source: Cell Death Dis. 2022 Oct 25;13(10):896. doi: 10.1038/s41419-022-05338-y (PMC9592618; doi:10.1038/s41419-022-05338-y)

FIG.2B

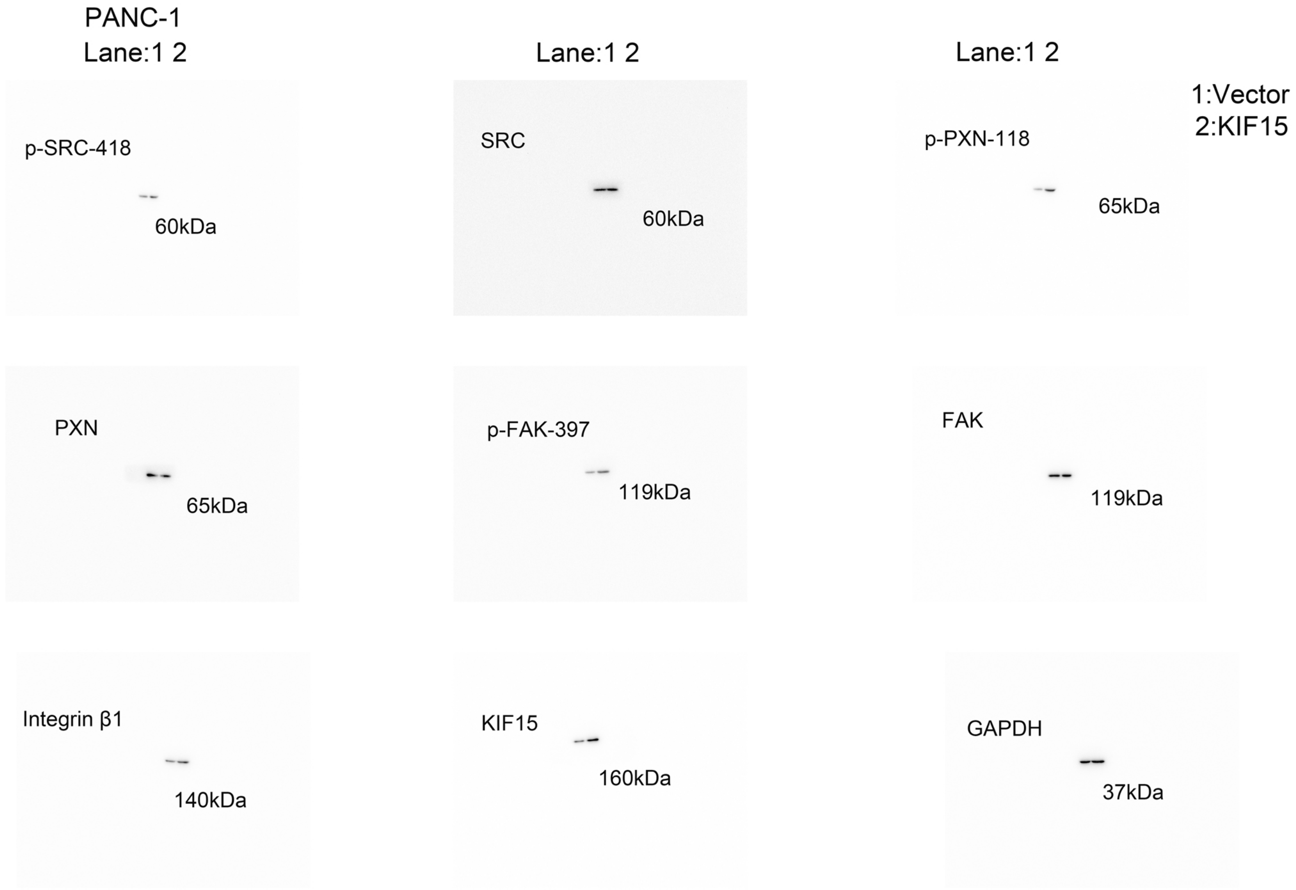
PANC-1
Lane:1 2
Lane:1 2
Lane:1 2
1:Vector
2:KIF15
p-SRC-418
60kDa
SRC
60kDa
p-PXN-118
65kDa
PXN
65kDa
p-FAK-397
119kDa
FAK
119kDa
Integrin β1
140kDa
KIF15
160kDa
GAPDH
37kDa

FIG.2B

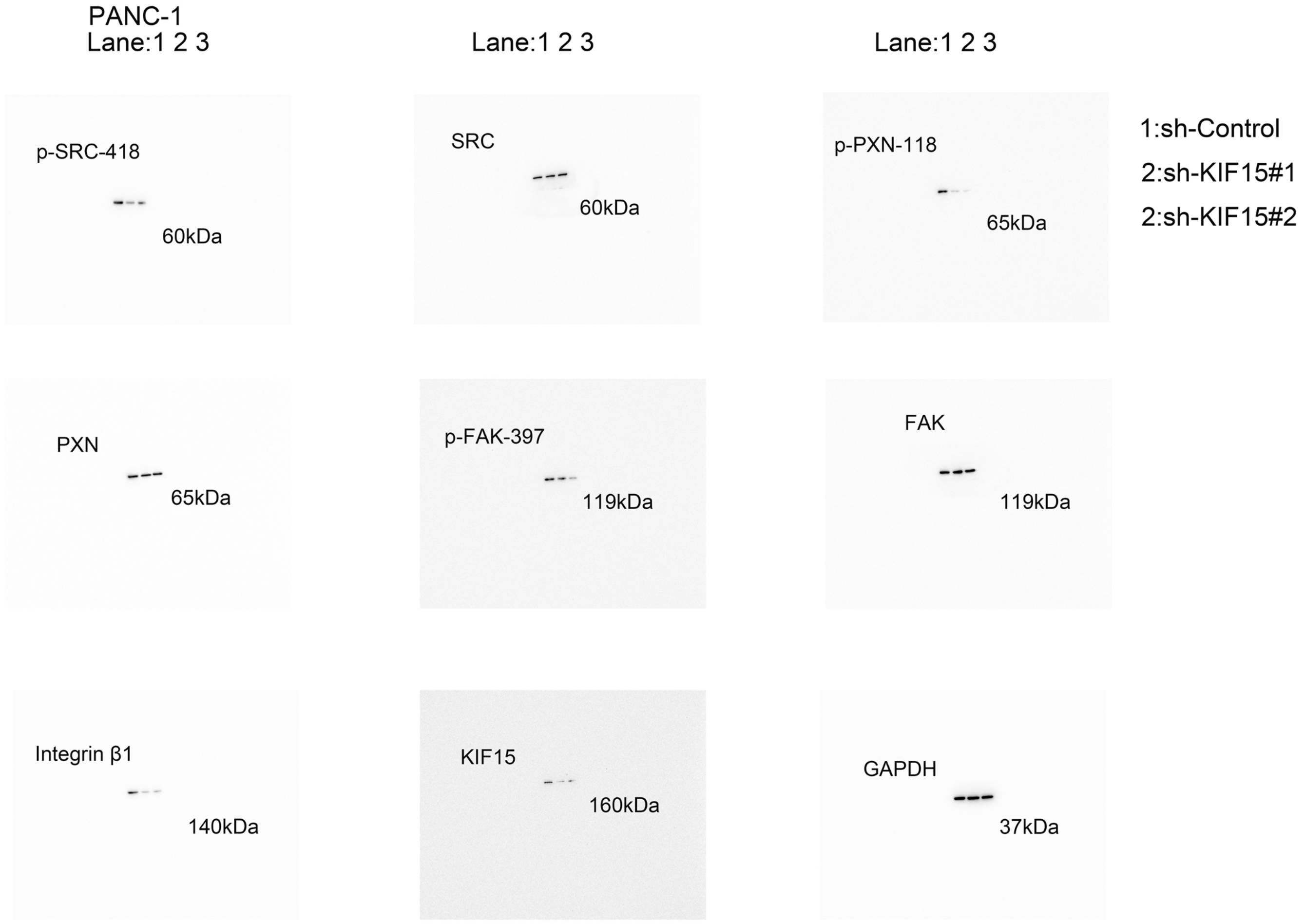
PANC-1
Lane:1 2 3
Lane:1 2 3
Lane:1 2 3
1:sh-Control
2:sh-KIF15#1
2:sh-KIF15#2
p-SRC-418
60kDa
SRC
60kDa
p-PXN-118
65kDa
PXN
65kDa
p-FAK-397
119kDa
FAK
119kDa
Integrin β1
140kDa
KIF15
160kDa
GAPDH
37kDa

FIG.2B

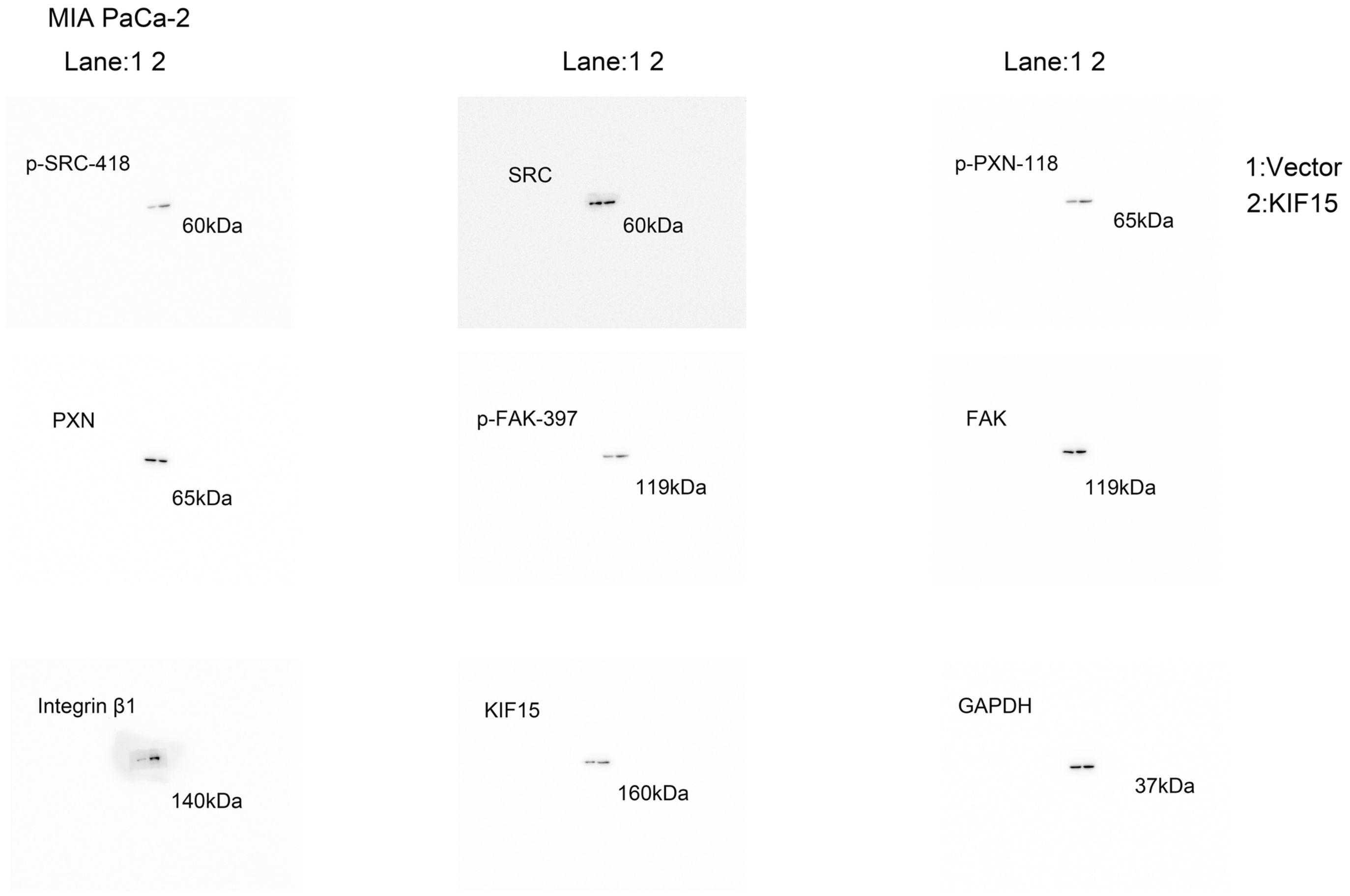
MIA PaCa-2
Lane:1 2
Lane:1 2
Lane:1 2
p-SRC-418
60kDa
SRC
60kDa
p-PXN-118
65kDa
1:Vector
2:KIF15
PXN
65kDa
p-FAK-397
119kDa
FAK
119kDa
Integrin β1
140kDa
KIF15
160kDa
GAPDH
37kDa

FIG.2B

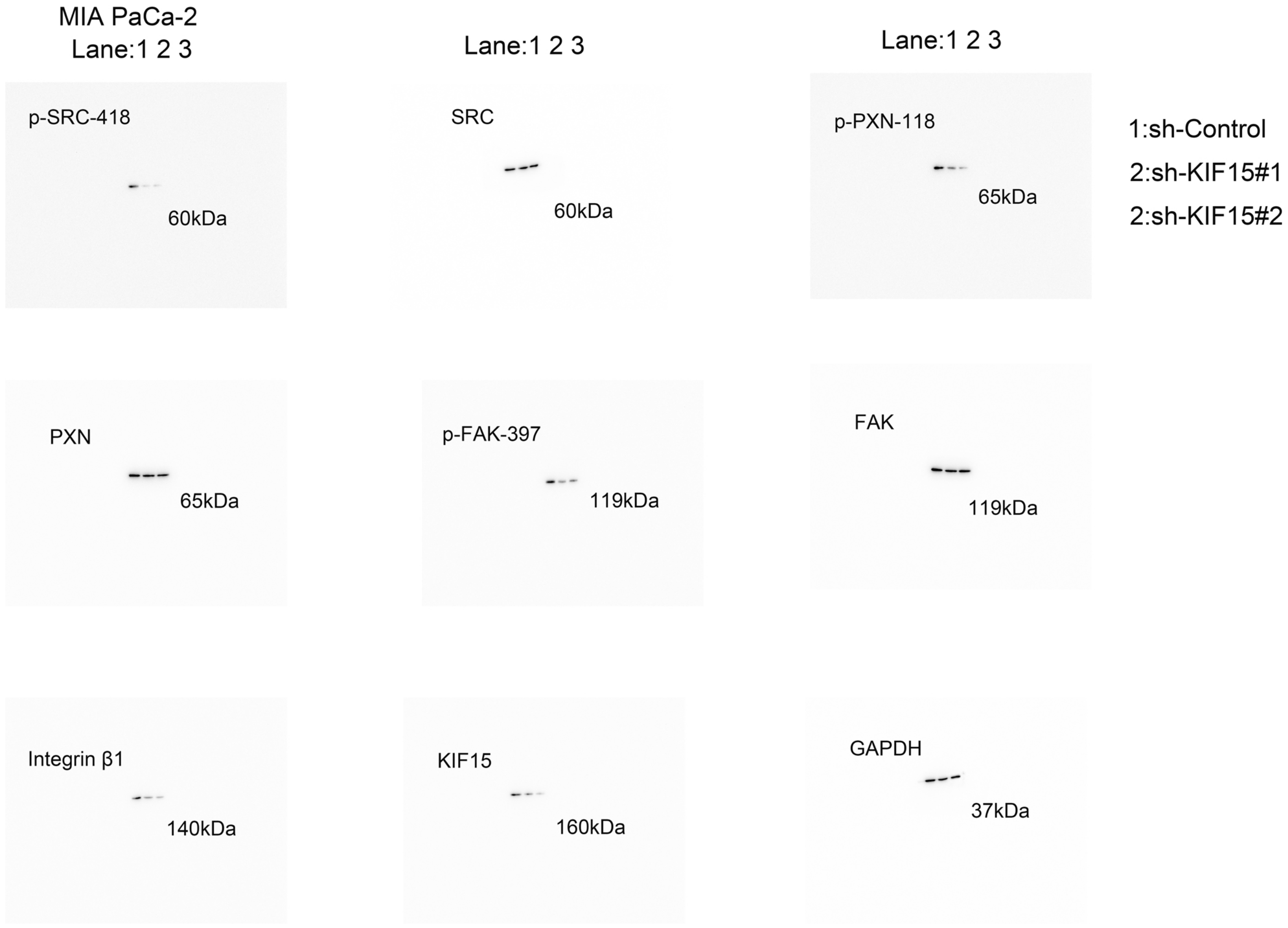
MIA PaCa-2
Lane:1 2 3
Lane:1 2 3
Lane:1 2 3
p-SRC-418
60kDa
SRC
60kDa
p-PXN-118
65kDa
1:sh-Control
2:sh-KIF15#1
2:sh-KIF15#2
PXN
65kDa
p-FAK-397
119kDa
FAK
119kDa
Integrin β1
140kDa
KIF15
160kDa
GAPDH
37kDa

FIG2D

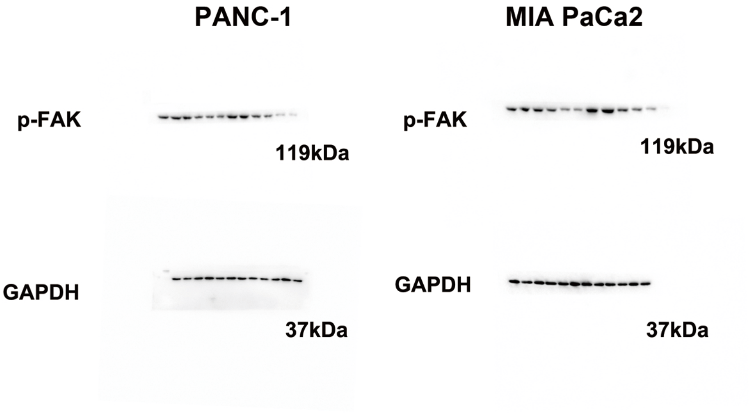
PANC-1
MIA PaCa2
p-FAK
119kDa
p-FAK
119kDa
GAPDH
37kDa
GAPDH
37kDa

FIG.3B

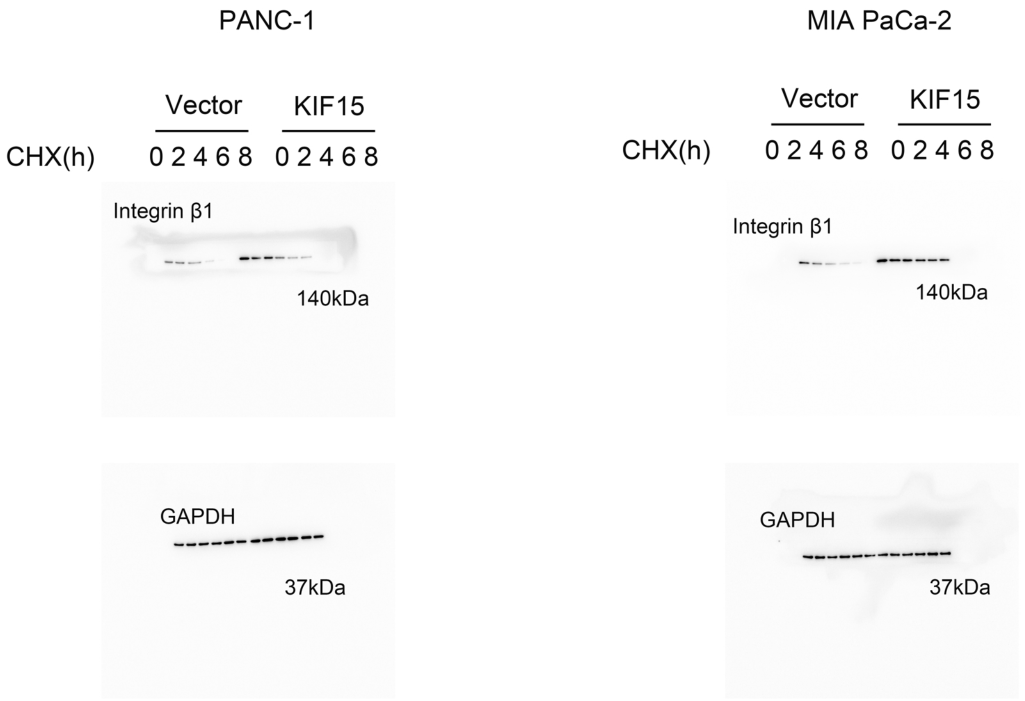

FIG.3C PANC-1

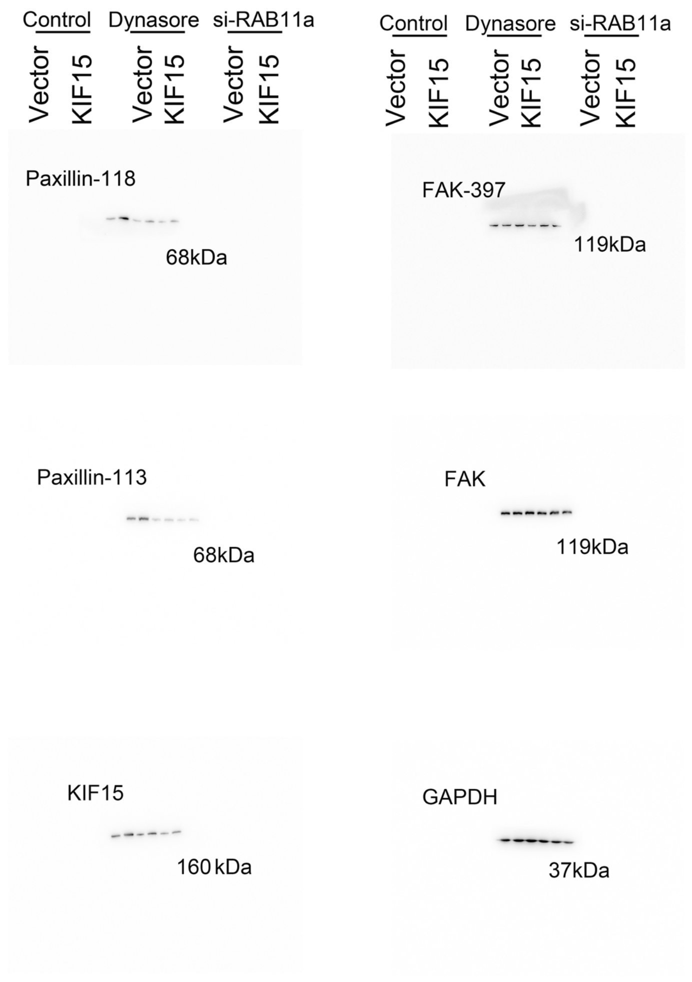

FIG.3C MIA PaCa-2

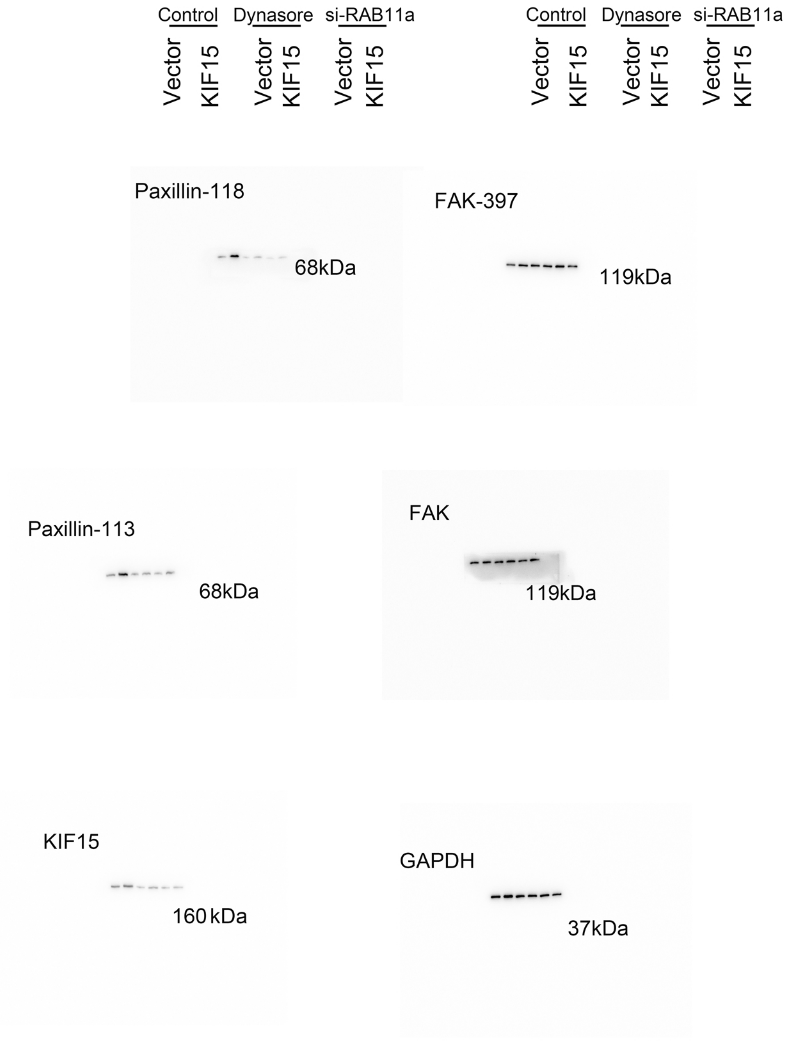

FIG.3D PANC-1

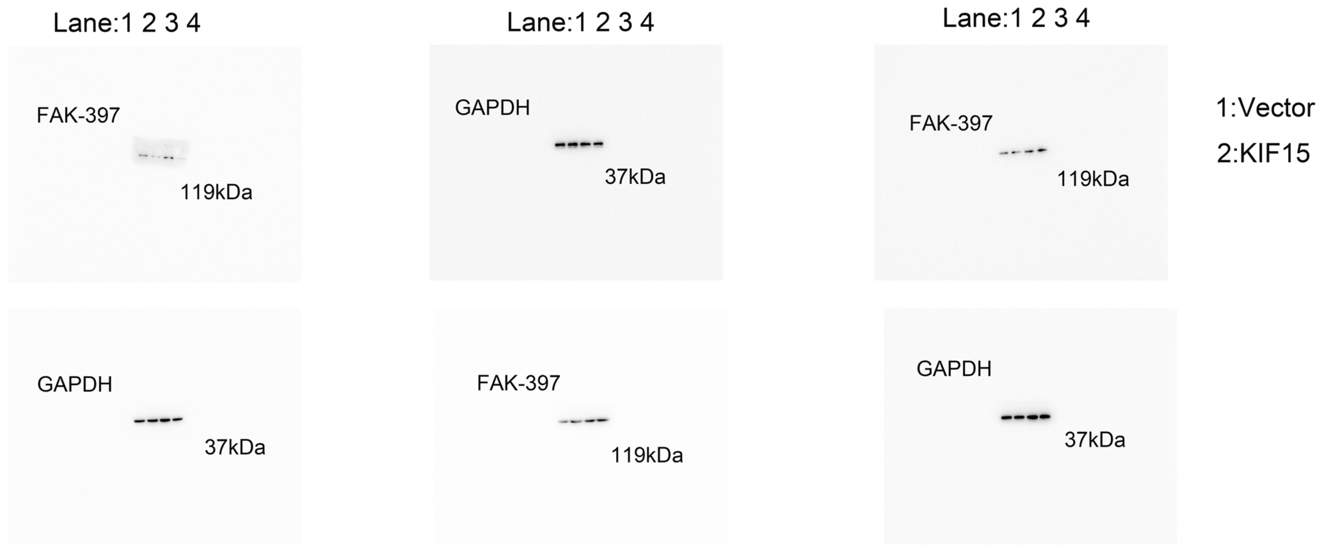

FIG.3D MIA PaCa-2

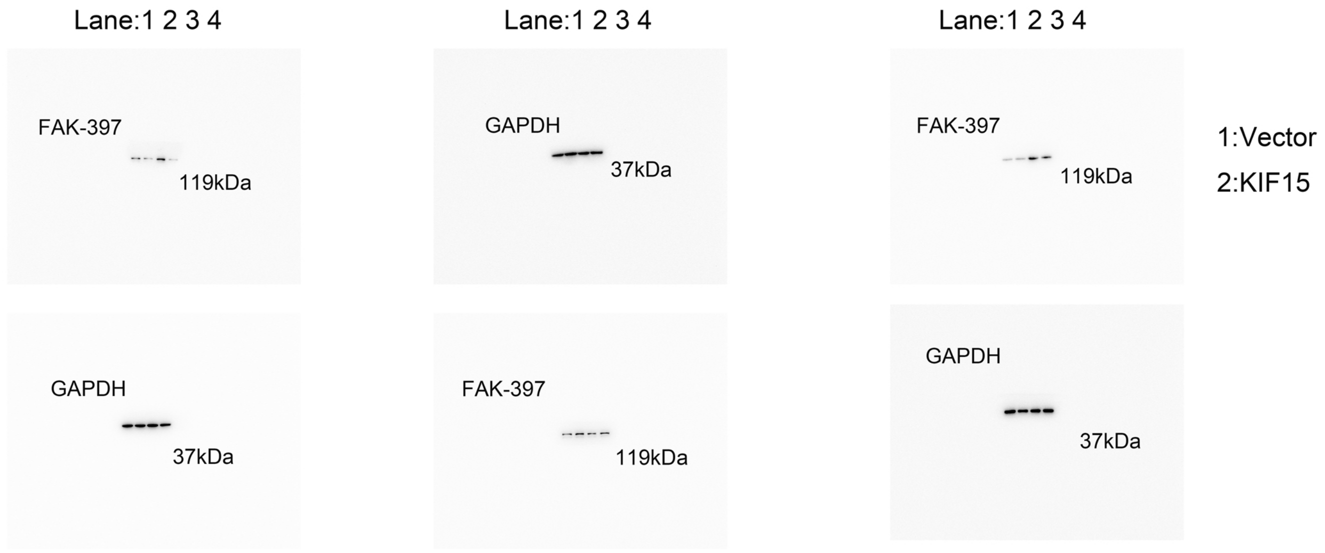

FIG.4C

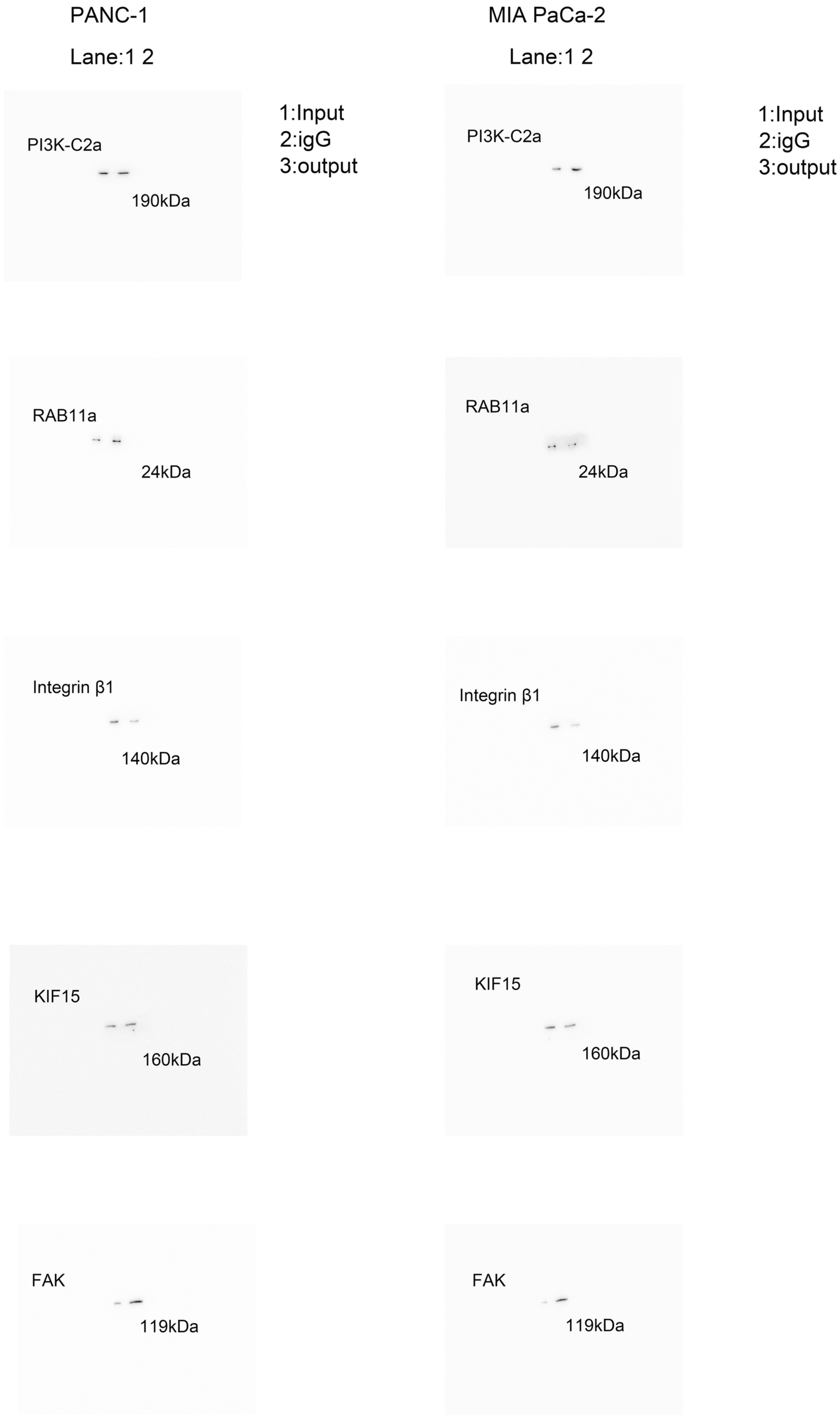
PANC-1
Lane:1 2
MIA PaCa-2
Lane:1 2
1:Input
2:igG
3:output
PI3K-C2a
190kDa
PI3K-C2a
190kDa
1:Input
2:igG
3:output
RAB11a
24kDa
RAB11a
24kDa
Integrin β1
140kDa
Integrin β1
140kDa
KIF15
160kDa
KIF15
160kDa
FAK
119kDa
FAK
119kDa

FIG.4E

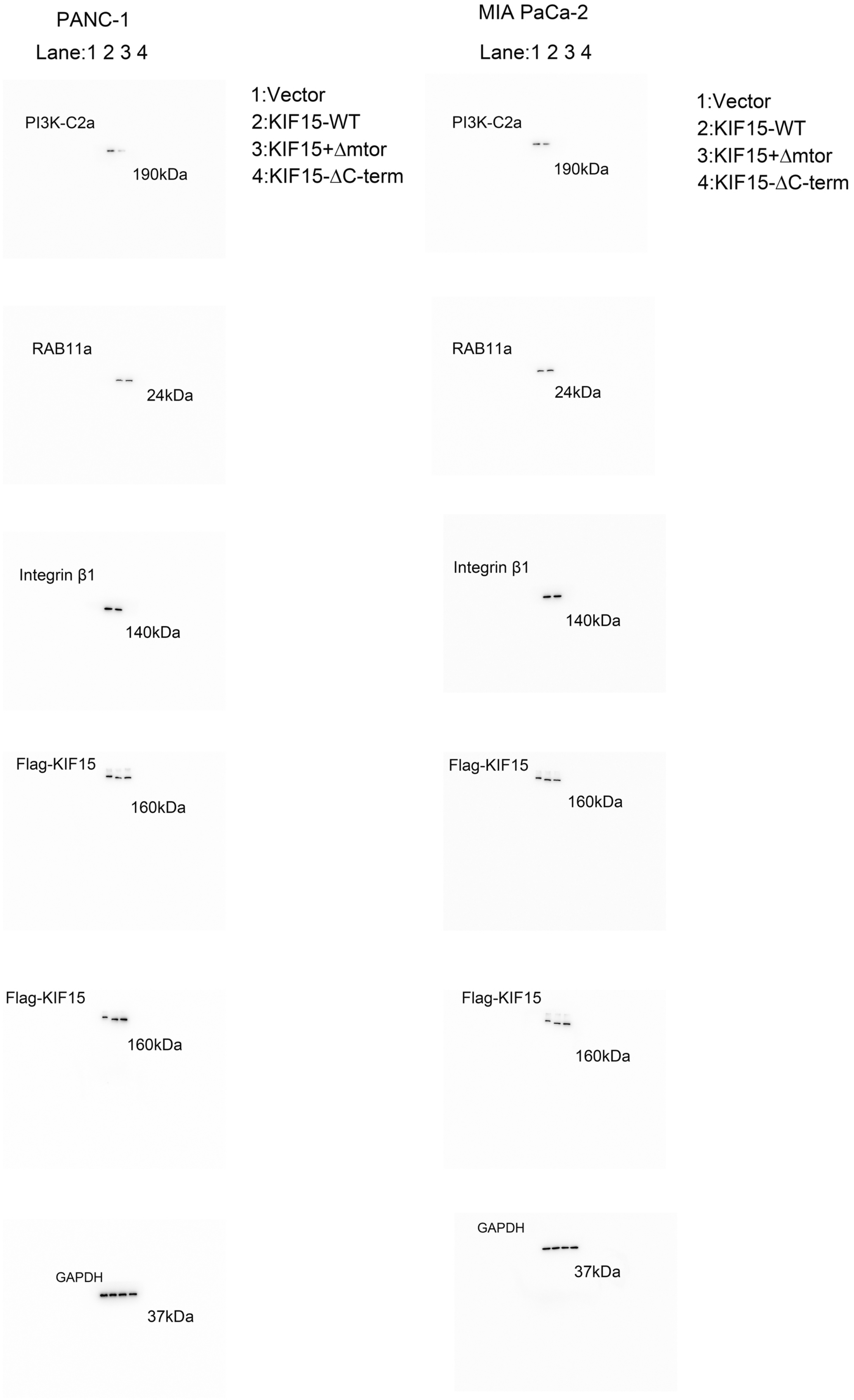
PANC-1
Lane:1 2 3 4
PI3K-C2a
190kDa
RAB11a
24kDa
Integrin β1
140kDa
Flag-KIF15
160kDa
Flag-KIF15
160kDa
GAPDH
37kDa
1:Vector
2:KIF15-WT
3:KIF15+Δmtor
4:KIF15-ΔC-term
MIA PaCa-2
Lane:1 2 3 4
PI3K-C2a
190kDa
RAB11a
24kDa
Integrin β1
140kDa
Flag-KIF15
160kDa
Flag-KIF15
160kDa
GAPDH
37kDa
1:Vector
2:KIF15-WT
3:KIF15+Δmtor
4:KIF15-ΔC-term

FIG.4F PANC-1

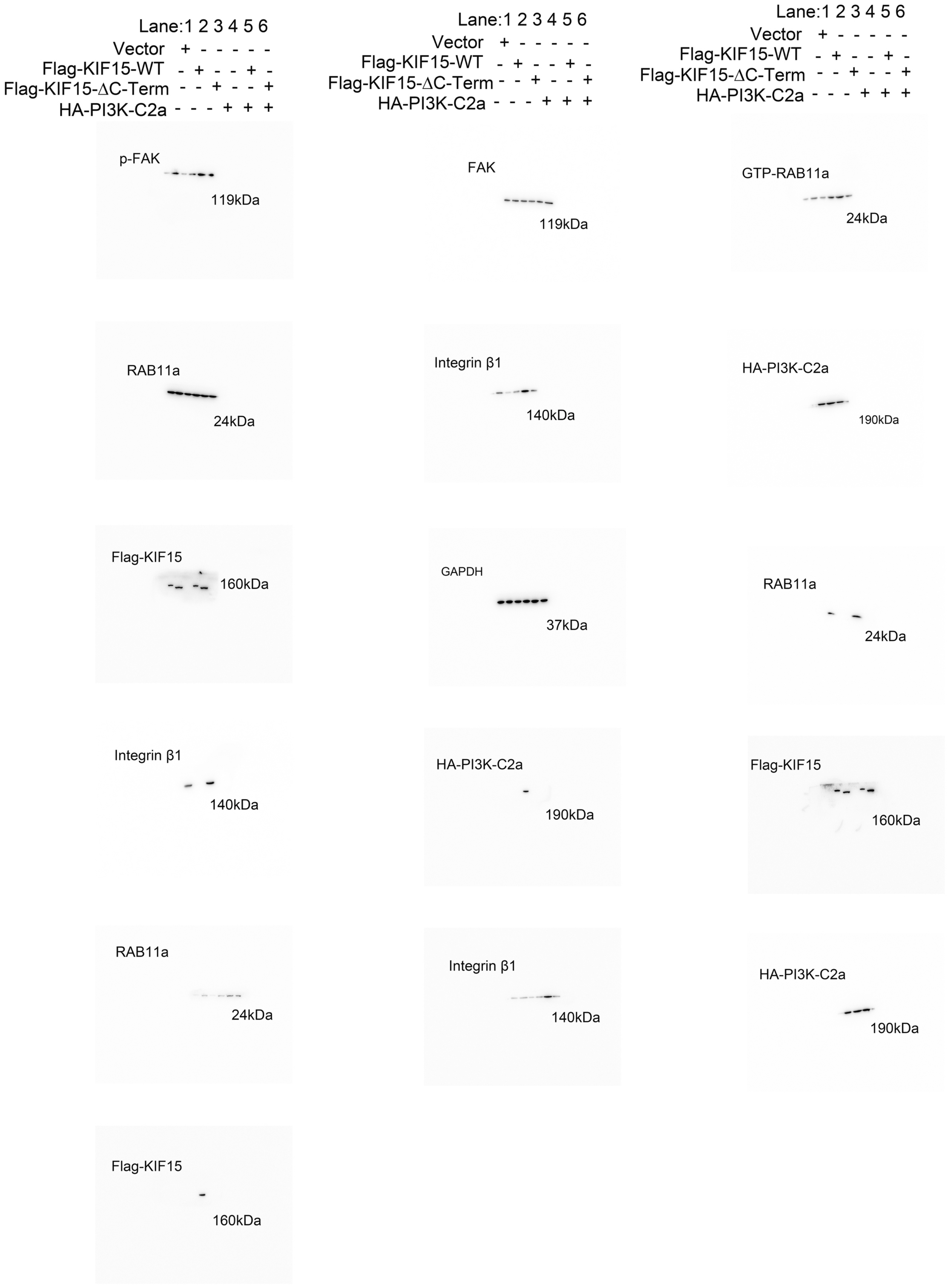
Lane:1 2 3 4 5 6
Vector + - - - - -
Flag-KIF15-WT - + - - + -
Flag-KIF15-ΔC-Term - - + - - +
HA-PI3K-C2a - - - + + +
p-FAK
119kDa
FAK
119kDa
GTP-RAB11a
24kDa
RAB11a
24kDa
Integrin β1
140kDa
HA-PI3K-C2a
190kDa
Flag-KIF15
160kDa
GAPDH
37kDa
RAB11a
24kDa
Integrin β1
140kDa
HA-PI3K-C2a
190kDa
Flag-KIF15
160kDa
RAB11a
24kDa
Integrin β1
140kDa
HA-PI3K-C2a
190kDa
Flag-KIF15
160kDa

FIG.4F MIA PaCa-2

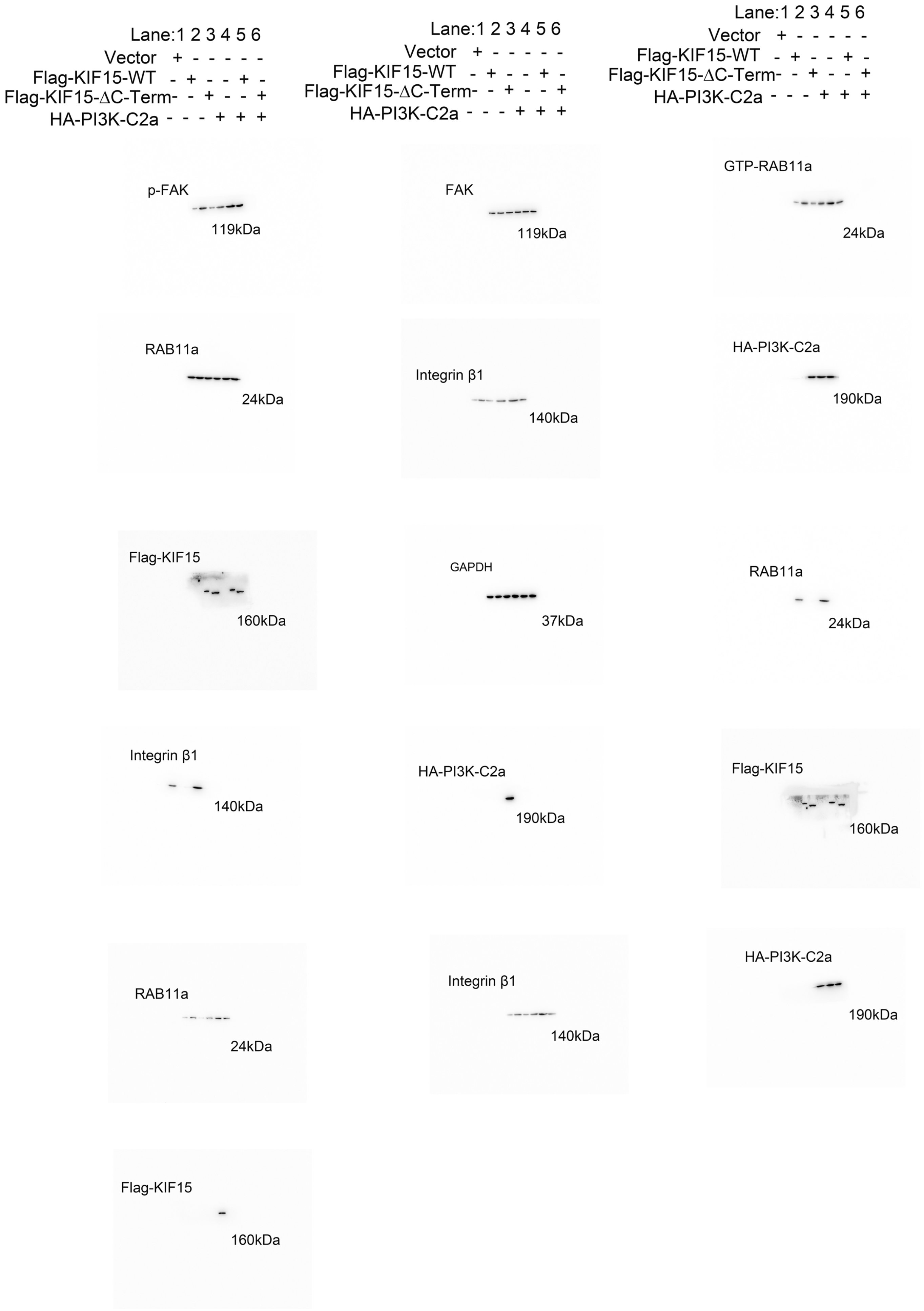

FIG.4H

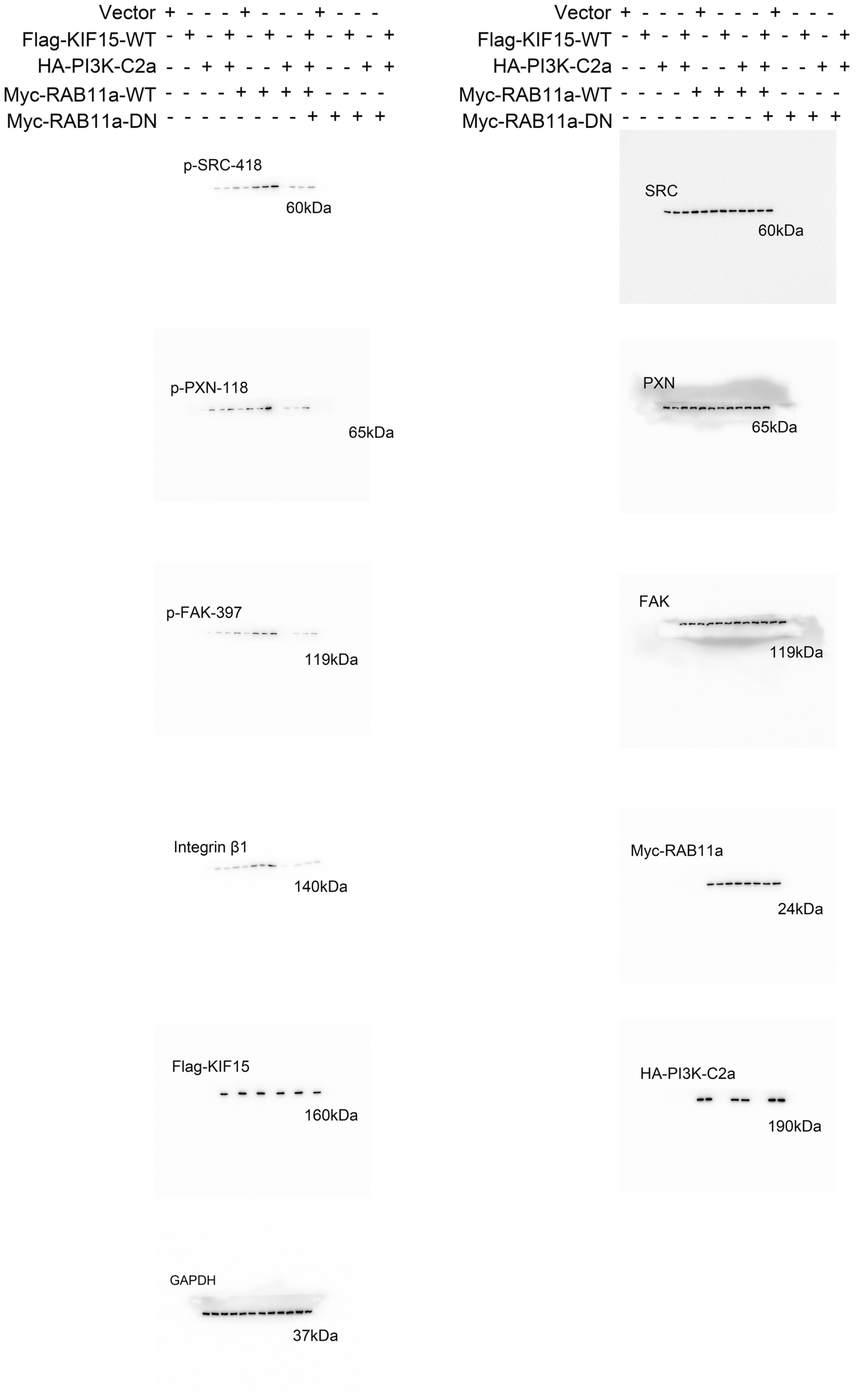
Vector + - - - + - - - + - - -
Flag-KIF15-WT - + - + - + - + - + - +
HA-PI3K-C2a - - + + - - + + - - + +
Myc-RAB11a-WT - - - - + + + + - - - -
Myc-RAB11a-DN - - - - - - - - + + + +
p-SRC-418
60kDa
p-PXN-118
65kDa
p-FAK-397
119kDa
Integrin β1
140kDa
Flag-KIF15
160kDa
GAPDH
37kDa
Vector + - - - + - - - + - - -
Flag-KIF15-WT - + - + - + - + - + - +
HA-PI3K-C2a - - + + - - + + - - + +
Myc-RAB11a-WT - - - - + + + + - - - -
Myc-RAB11a-DN - - - - - - - - + + + +
SRC
60kDa
PXN
65kDa
FAK
119kDa
Myc-RAB11a
24kDa
HA-PI3K-C2a
190kDa

FIG.5C

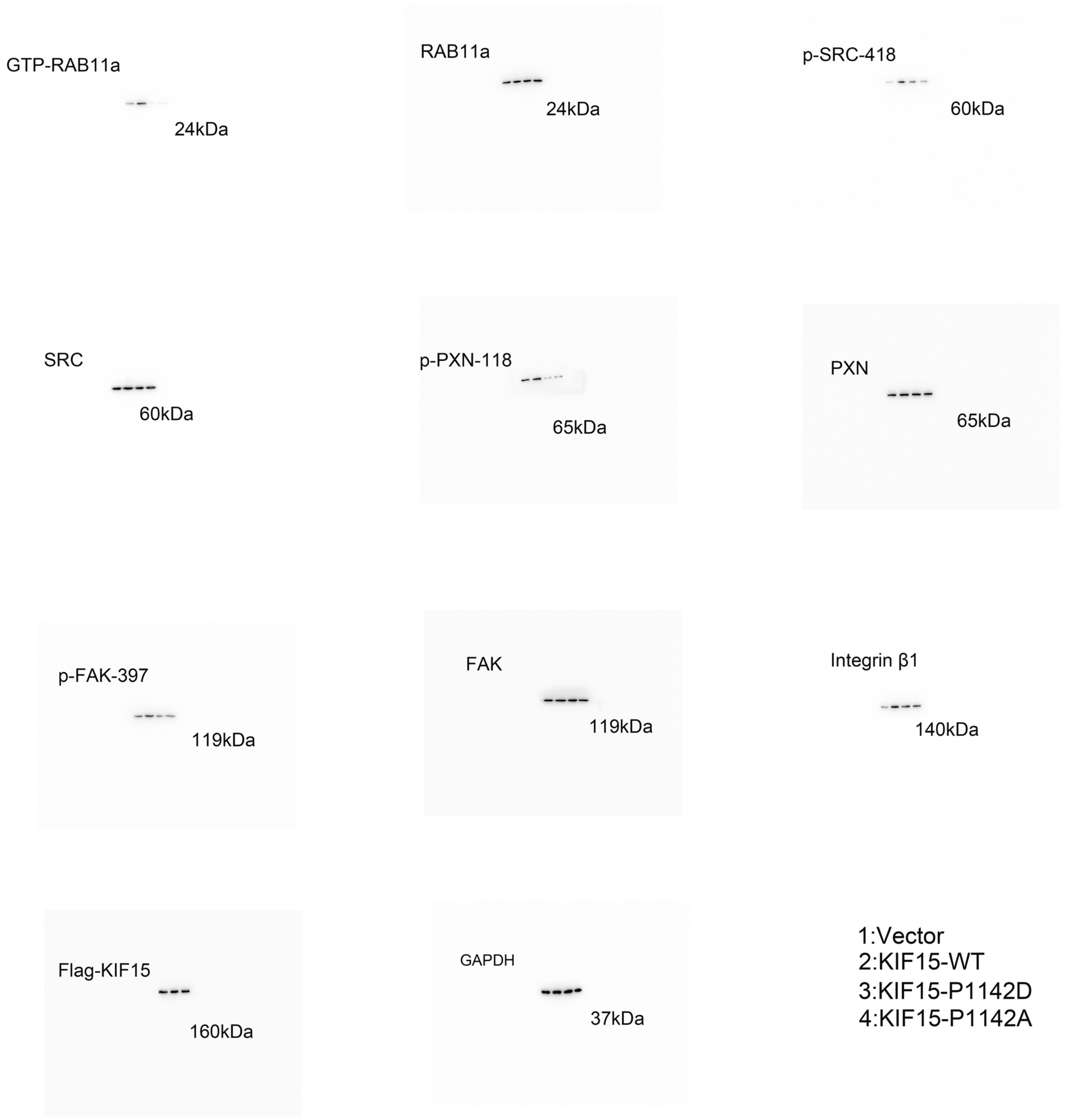
GTP-RAB11a
24kDa
RAB11a
24kDa
p-SRC-418
60kDa
SRC
60kDa
p-PXN-118
65kDa
PXN
65kDa
p-FAK-397
119kDa
FAK
119kDa
Integrin β1
140kDa
Flag-KIF15
160kDa
GAPDH
37kDa
1:Vector
2:KIF15-WT
3:KIF15-P1142D
4:KIF15-P1142A

FIG.5C

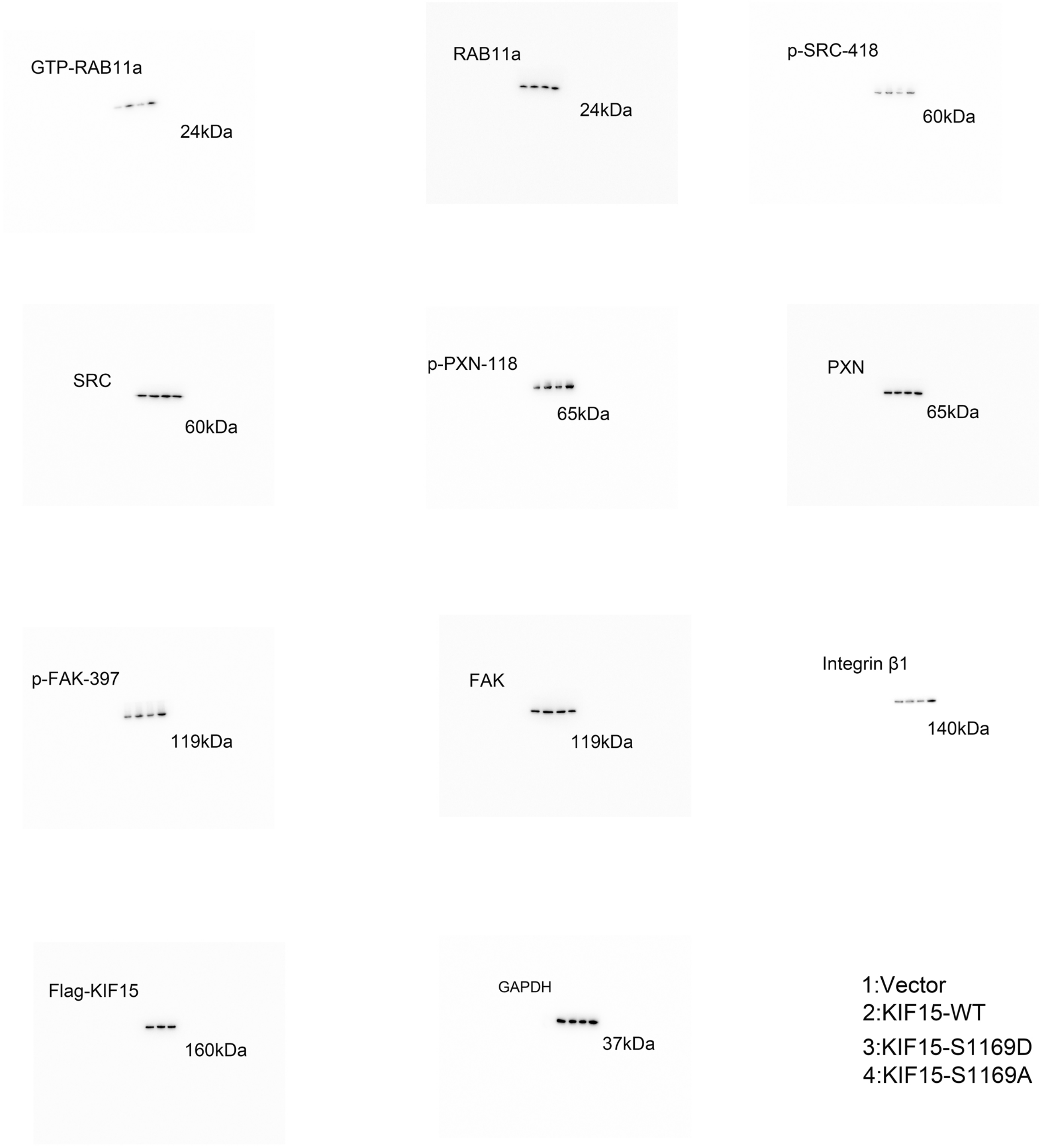
GTP-RAB11a
24kDa
RAB11a
24kDa
p-SRC-418
60kDa
SRC
60kDa
p-PXN-118
65kDa
PXN
65kDa
p-FAK-397
119kDa
FAK
119kDa
Integrin β1
140kDa
Flag-KIF15
160kDa
GAPDH
37kDa
1:Vector
2:KIF15-WT
3:KIF15-S1169D
4:KIF15-S1169A

FIG.5C

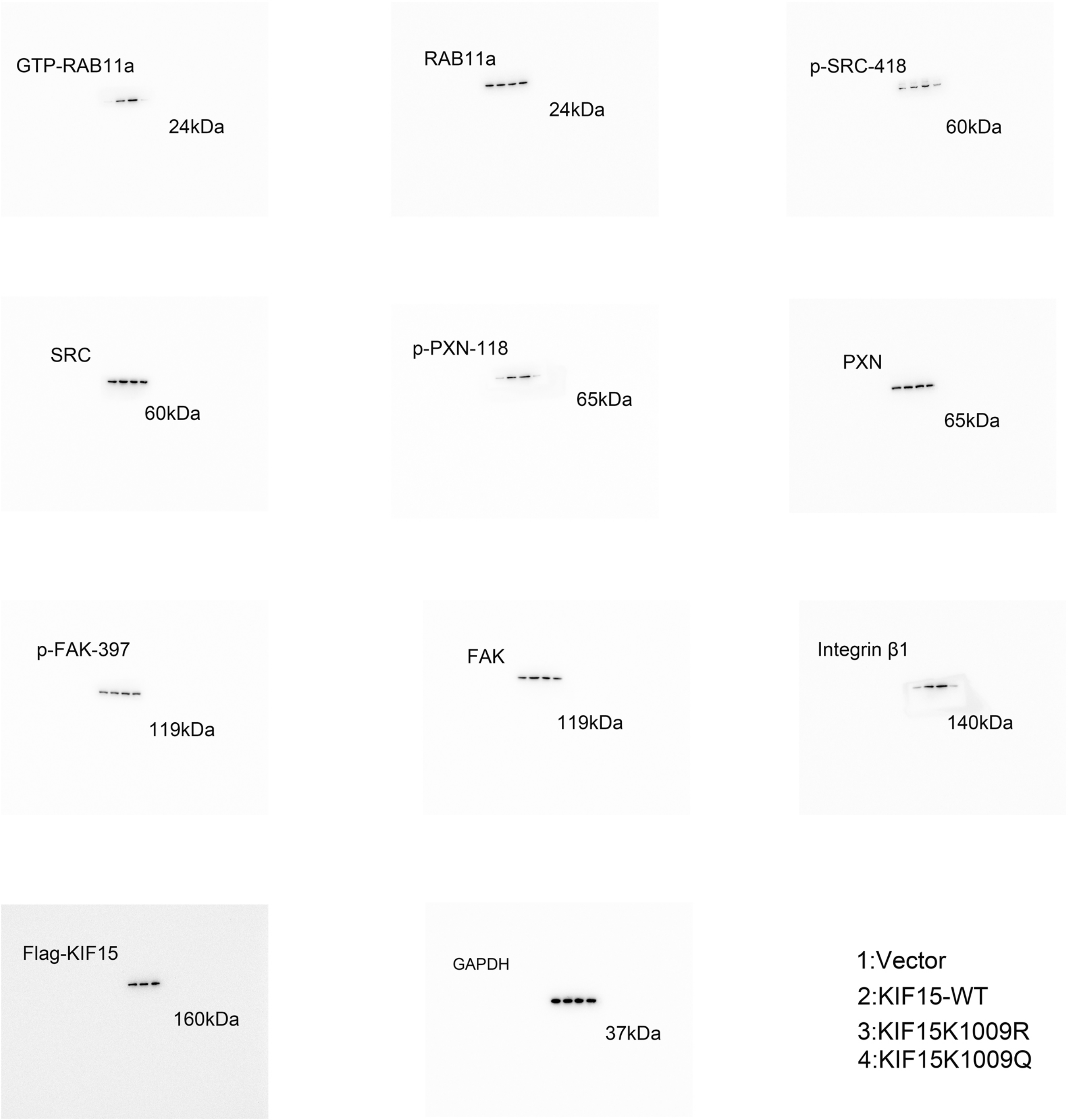
GTP-RAB11a
24kDa
RAB11a
24kDa
p-SRC-418
60kDa
SRC
60kDa
p-PXN-118
65kDa
PXN
65kDa
p-FAK-397
119kDa
FAK
119kDa
Integrin β1
140kDa
Flag-KIF15
160kDa
GAPDH
37kDa
1:Vector
2:KIF15-WT
3:KIF15K1009R
4:KIF15K1009Q

FIG.5E

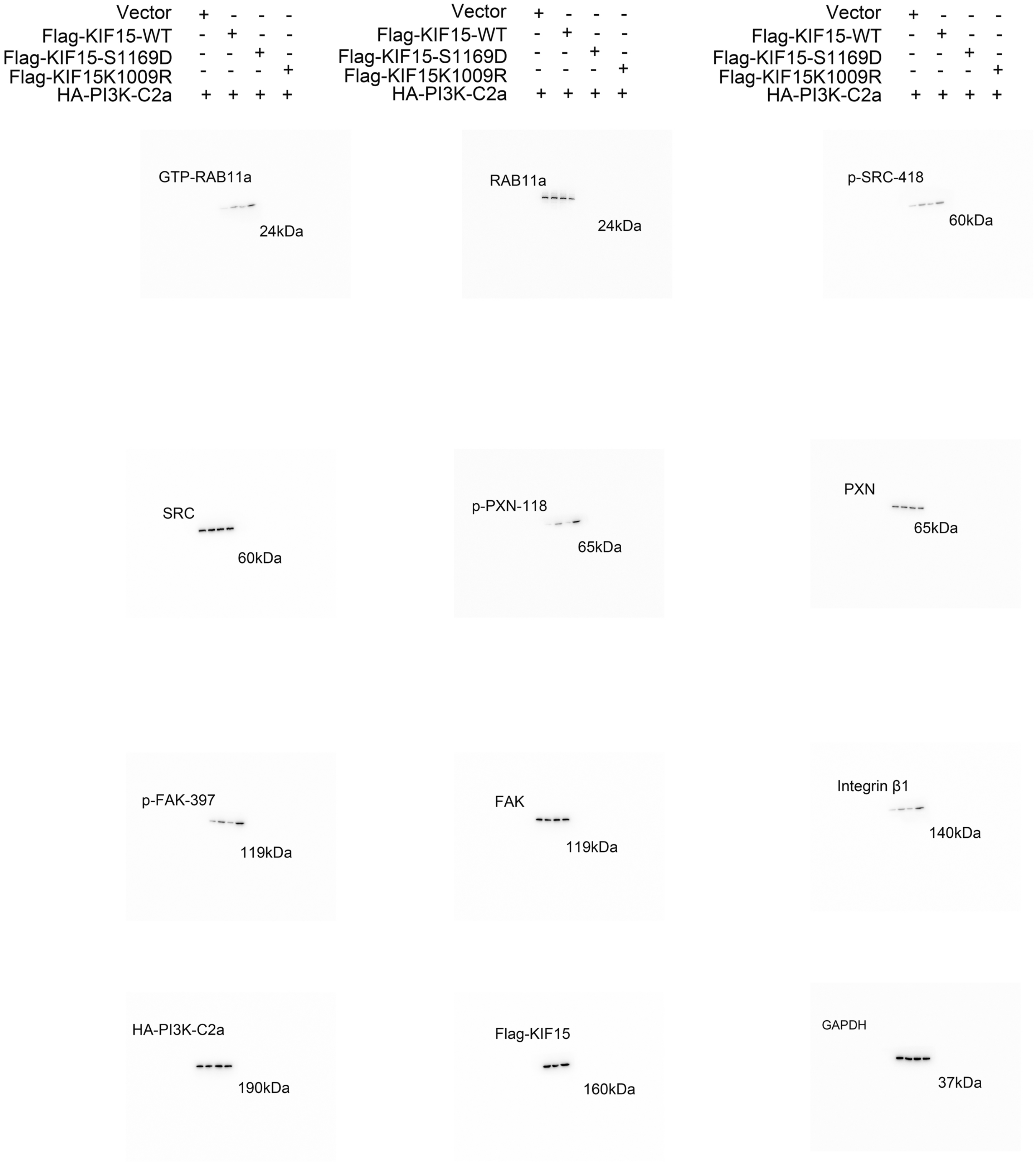
Vector + - - -
Flag-KIF15-WT - + - -
Flag-KIF15-S1169D - - + -
Flag-KIF15K1009R - - - +
HA-PI3K-C2a + + + +
GTP-RAB11a
24kDa
RAB11a
24kDa
p-SRC-418
60kDa
SRC
60kDa
p-PXN-118
65kDa
PXN
65kDa
p-FAK-397
119kDa
FAK
119kDa
Integrin β1
140kDa
HA-PI3K-C2a
190kDa
Flag-KIF15
160kDa
GAPDH
37kDa

FIG.5E

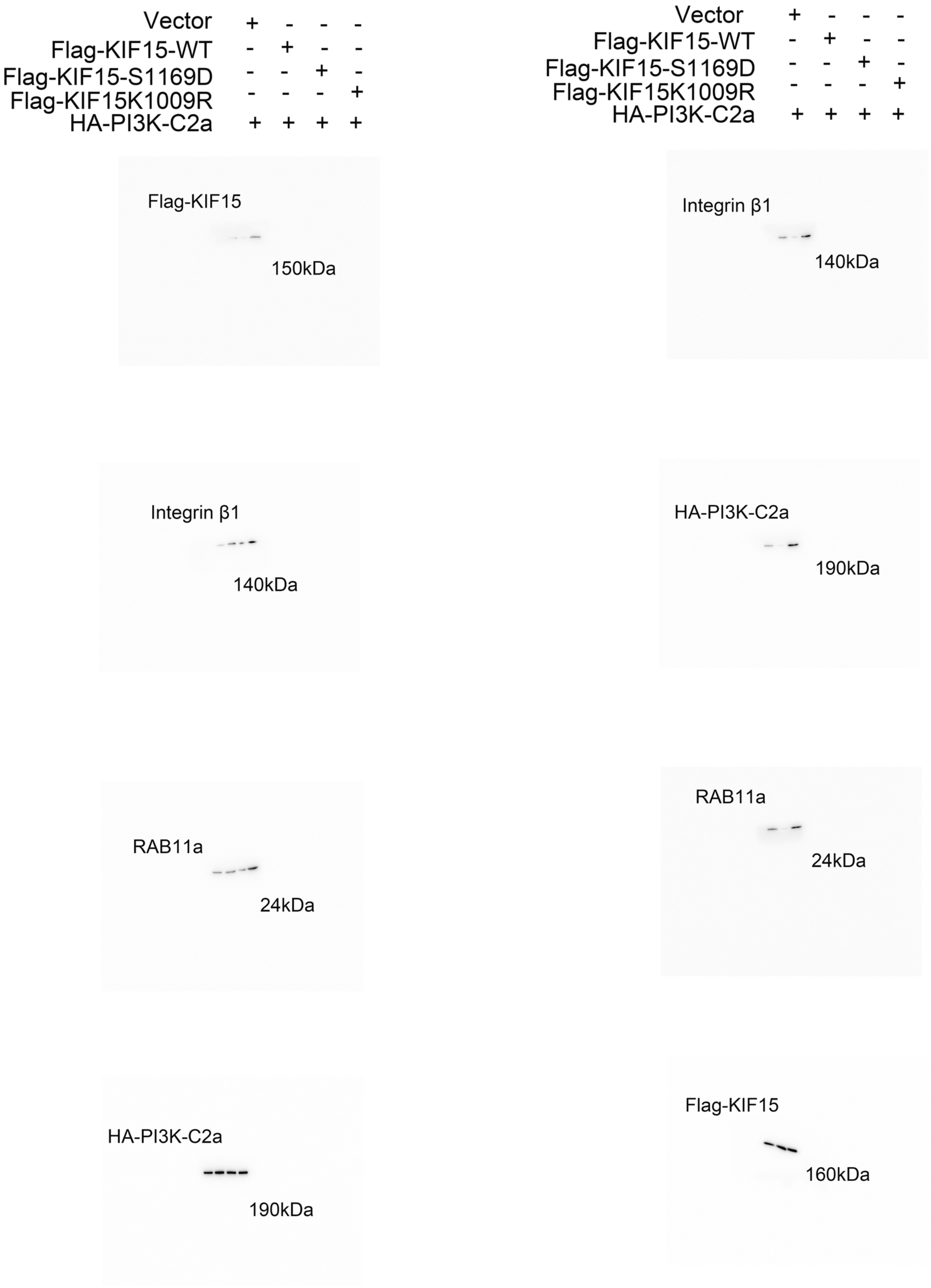

Vector + - - -
Flag-KIF15-WT - + - -
Flag-KIF15-S1169D - - + -
Flag-KIF15K1009R - - - +
HA-PI3K-C2a + + + +
Flag-KIF15
150kDa
Integrin β1
140kDa
RAB11a
24kDa
HA-PI3K-C2a
190kDa
Vector + - - -
Flag-KIF15-WT - + - -
Flag-KIF15-S1169D - - + -
Flag-KIF15K1009R - - - +
HA-PI3K-C2a + + + +
Integrin β1
140kDa
HA-PI3K-C2a
190kDa
RAB11a
24kDa
Flag-KIF15
160kDa

FIG.5F

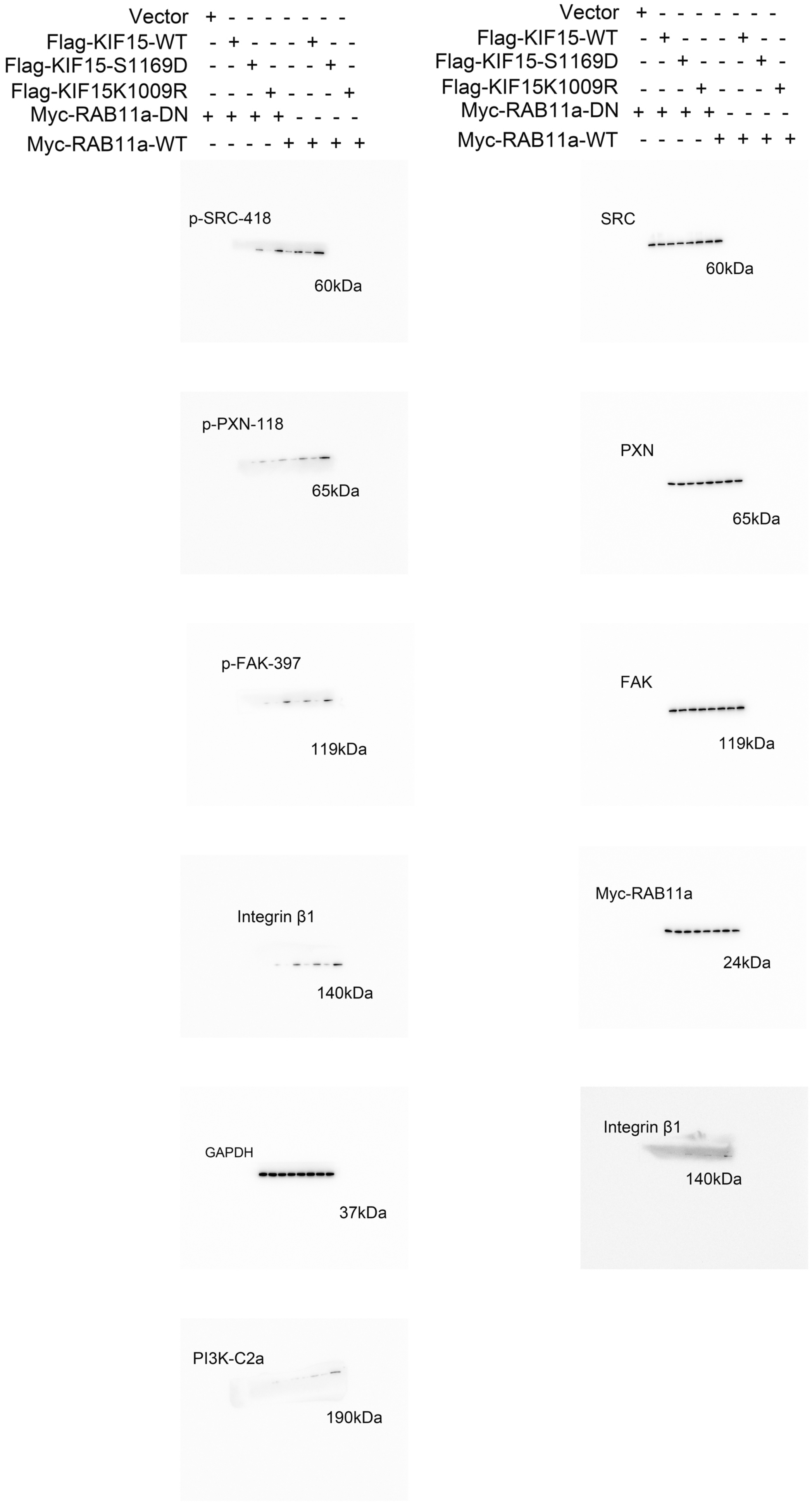

Vector + - - - - - - -
Flag-KIF15-WT - + - - - - + - -
Flag-KIF15-S1169D - - + - - - - + -
Flag-KIF15K1009R - - - + - - - - +
Myc-RAB11a-DN + + + + - - - -
Myc-RAB11a-WT - - - - + + + +
p-SRC-418
60kDa
p-PXN-118
65kDa
p-FAK-397
119kDa
Integrin β1
140kDa
GAPDH
37kDa
PI3K-C2a
190kDa
Vector + - - - - - - -
Flag-KIF15-WT - + - - - + - -
Flag-KIF15-S1169D - - + - - - - + -
Flag-KIF15K1009R - - - + - - - - +
Myc-RAB11a-DN + + + + - - - -
Myc-RAB11a-WT - - - - + + + +
SRC
60kDa
PXN
65kDa
FAK
119kDa
Myc-RAB11a
24kDa
Integrin β1
140kDa

FIG.6A

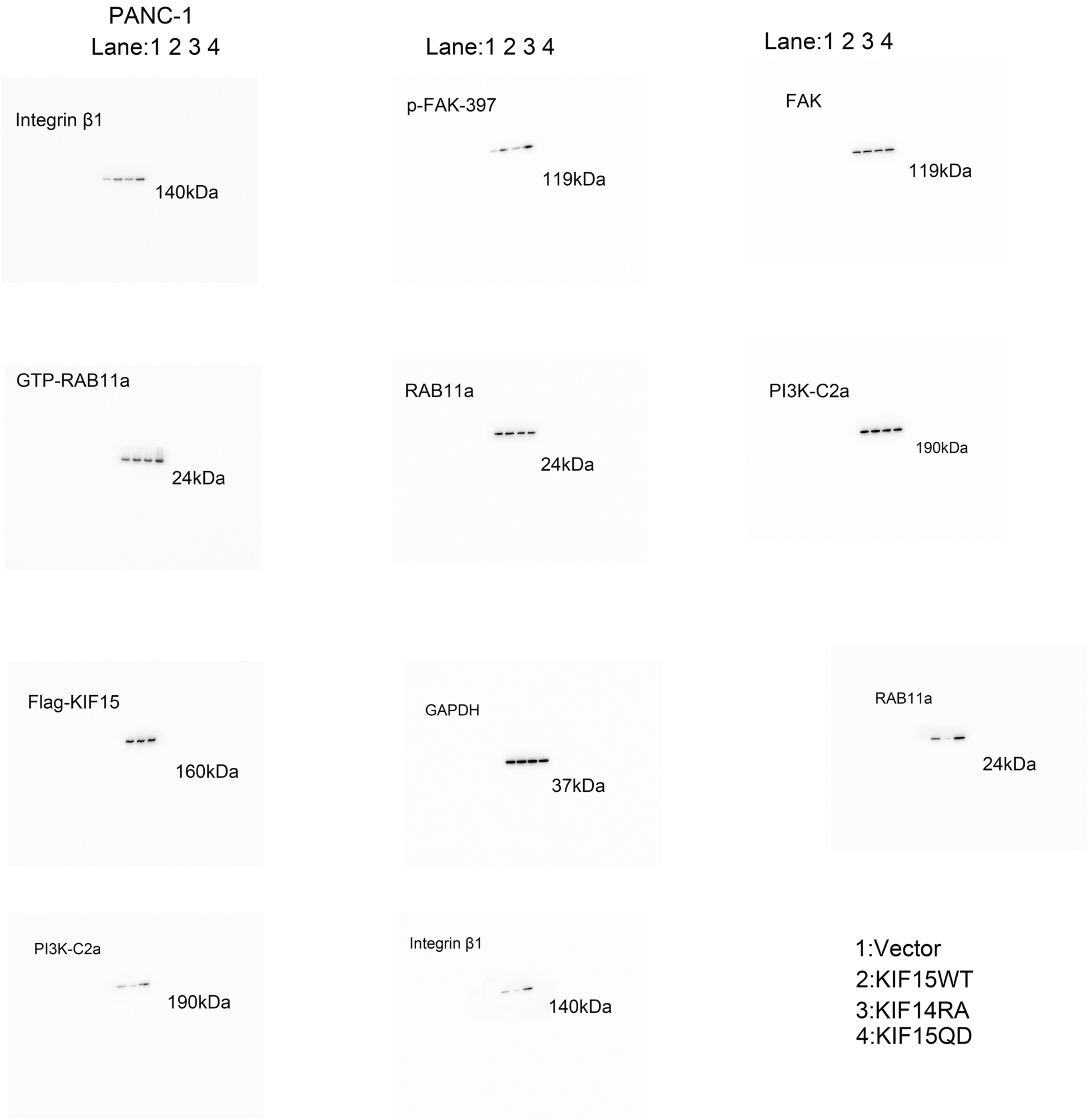
PANC-1
Lane:1 2 3 4
Lane:1 2 3 4
Lane:1 2 3 4
Integrin β1
140kDa
p-FAK-397
119kDa
FAK
119kDa
GTP-RAB11a
24kDa
RAB11a
24kDa
PI3K-C2a
190kDa
Flag-KIF15
160kDa
GAPDH
37kDa
RAB11a
24kDa
PI3K-C2a
190kDa
Integrin β1
140kDa
1:Vector
2:KIF15WT
3:KIF14RA
4:KIF15QD

FIG.6A

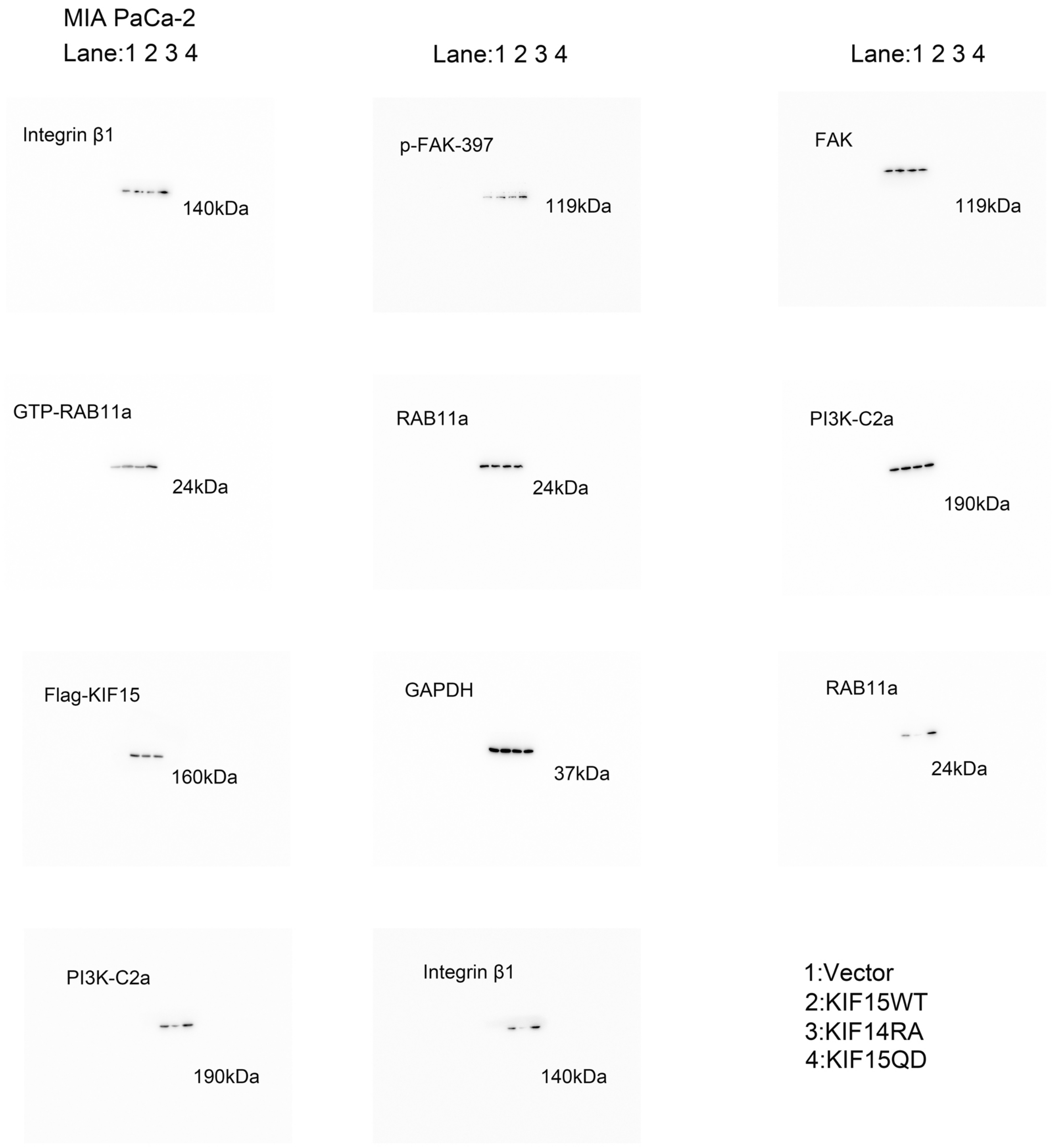
MIA PaCa-2
Lane:1 2 3 4
Lane:1 2 3 4
Lane:1 2 3 4
Integrin β1
140kDa
p-FAK-397
119kDa
FAK
119kDa
GTP-RAB11a
24kDa
RAB11a
24kDa
PI3K-C2a
190kDa
Flag-KIF15
160kDa
GAPDH
37kDa
RAB11a
24kDa
PI3K-C2a
190kDa
Integrin β1
140kDa
1:Vector
2:KIF15WT
3:KIF14RA
4:KIF15QD

FIG.6B

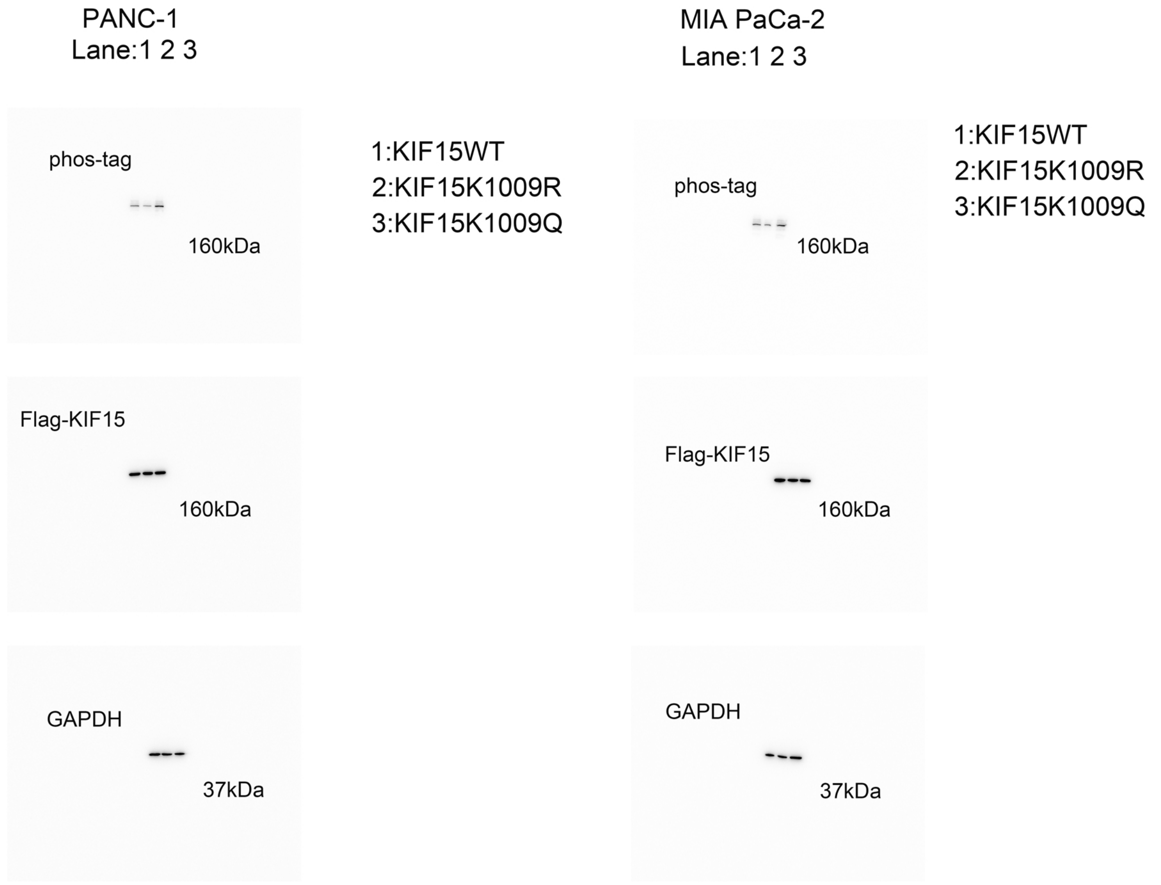
PANC-1
Lane:1 2 3
phos-tag
160kDa
Flag-KIF15
160kDa
GAPDH
37kDa
1:KIF15WT
2:KIF15K1009R
3:KIF15K1009Q
MIA PaCa-2
Lane:1 2 3
phos-tag
160kDa
Flag-KIF15
160kDa
GAPDH
37kDa
1:KIF15WT
2:KIF15K1009R
3:KIF15K1009Q

FIG.6C

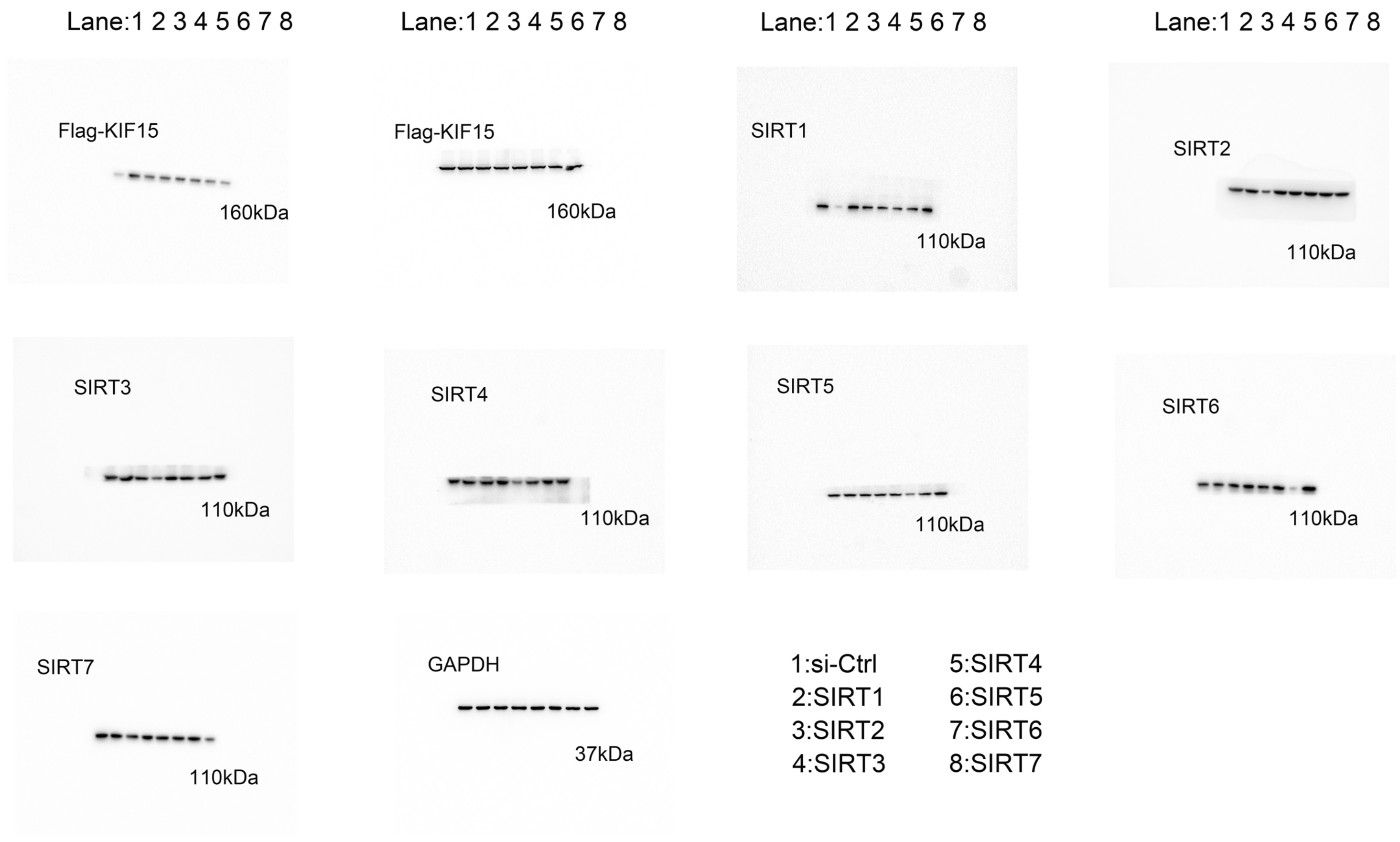
Lane:1 2 3 4 5 6 7 8
Flag-KIF15
160kDa
Lane:1 2 3 4 5 6 7 8
Flag-KIF15
160kDa
Lane:1 2 3 4 5 6 7 8
SIRT1
110kDa
Lane:1 2 3 4 5 6 7 8
SIRT2
110kDa
SIRT3
110kDa
SIRT4
110kDa
SIRT5
110kDa
SIRT6
110kDa
SIRT7
110kDa
GAPDH
37kDa
1:si-Ctrl
2:SIRT1
3:SIRT2
4:SIRT3
5:SIRT4
6:SIRT5
7:SIRT6
8:SIRT7

FIG.6F

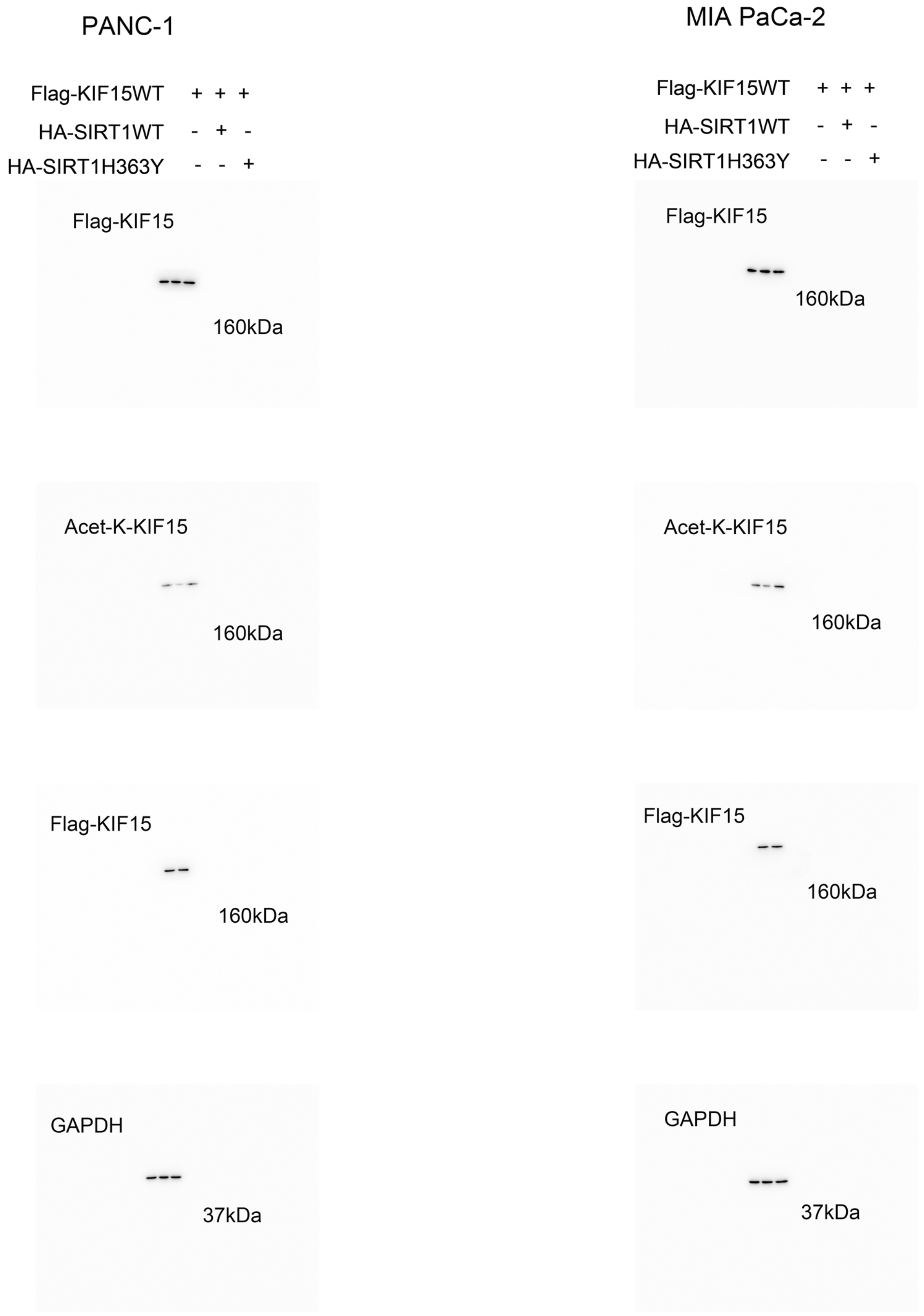
PANC-1
Flag-KIF15WT + + +
HA-SIRT1WT - + -
HA-SIRT1H363Y - - +
Flag-KIF15
160kDa
Acet-K-KIF15
160kDa
Flag-KIF15
160kDa
GAPDH
37kDa
MIA PaCa-2
Flag-KIF15WT + + +
HA-SIRT1WT - + -
HA-SIRT1H363Y - - +
Flag-KIF15
160kDa
Acet-K-KIF15
160kDa
Flag-KIF15
160kDa
GAPDH
37kDa

FIG.6G

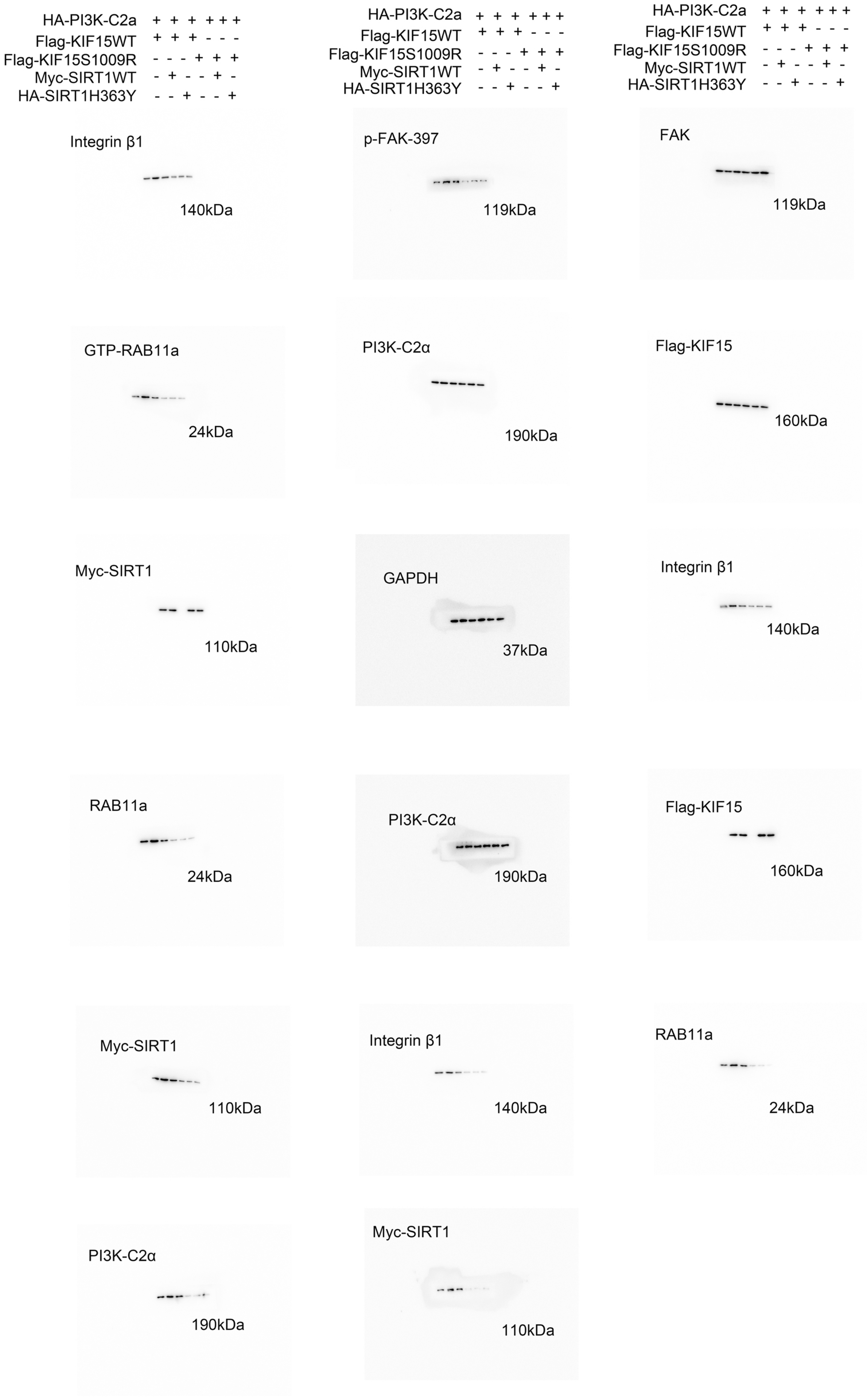

HA-PI3K-C2a + + + + + +
Flag-KIF15WT + + + - - -
Flag-KIF15S1009R - - - + + +
Myc-SIRT1WT - + - - + -
HA-SIRT1H363Y - - + - - +
Integrin β1
140kDa
p-FAK-397
119kDa
FAK
119kDa
GTP-RAB11a
24kDa
PI3K-C2α
190kDa
Flag-KIF15
160kDa
Myc-SIRT1
110kDa
GAPDH
37kDa
Integrin β1
140kDa
RAB11a
24kDa
PI3K-C2α
190kDa
Flag-KIF15
160kDa
Myc-SIRT1
110kDa
Integrin β1
140kDa
RAB11a
24kDa
PI3K-C2α
190kDa
Myc-SIRT1
110kDa

FIG.7C PANC-1

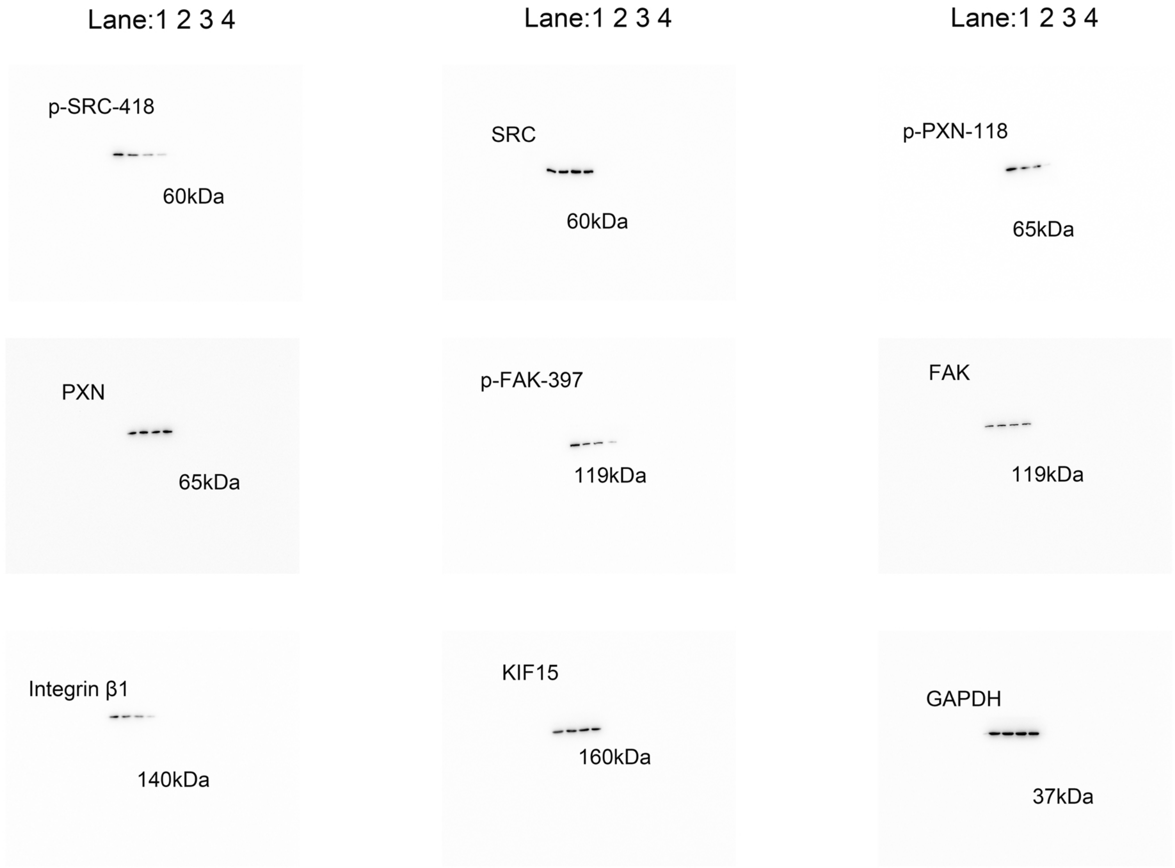

FIG.7C MIA PaCa-2

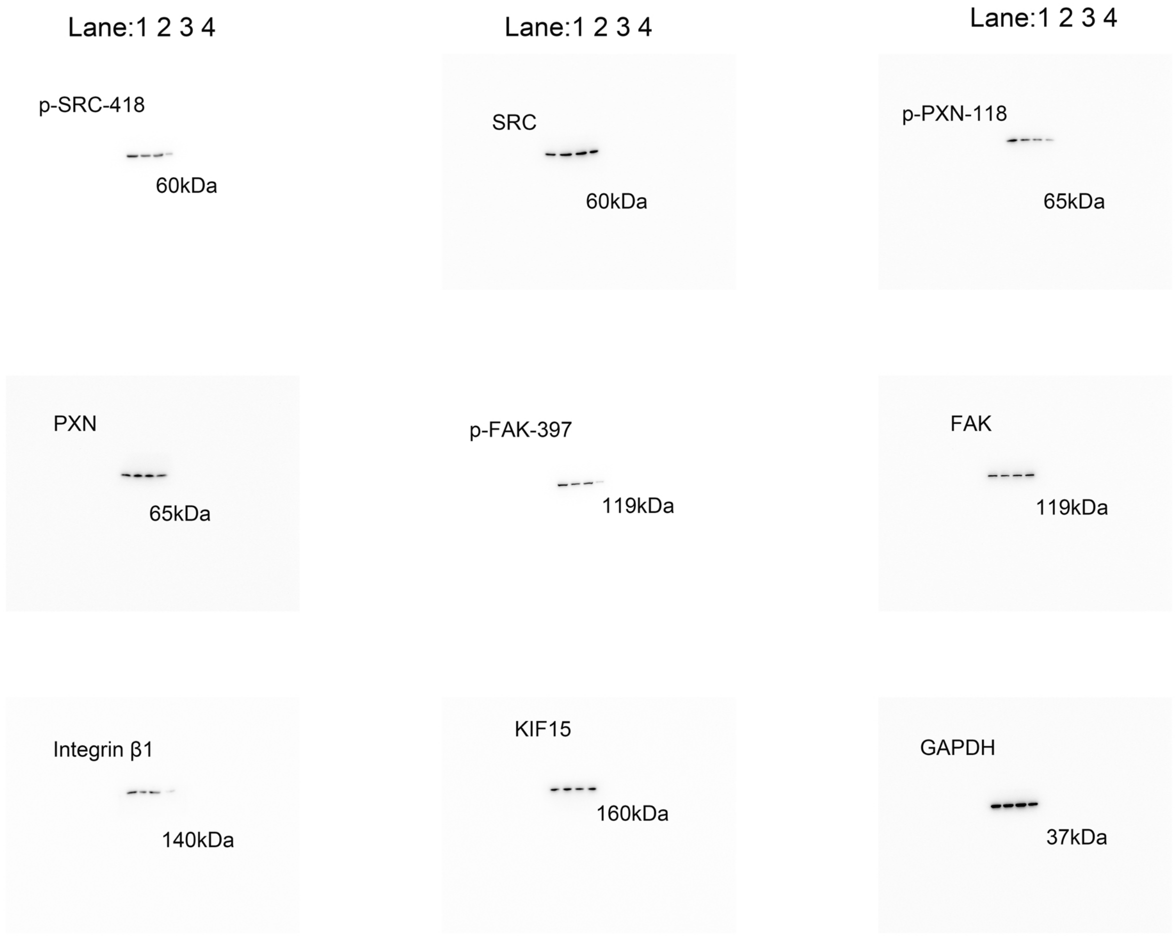

FIG.7C

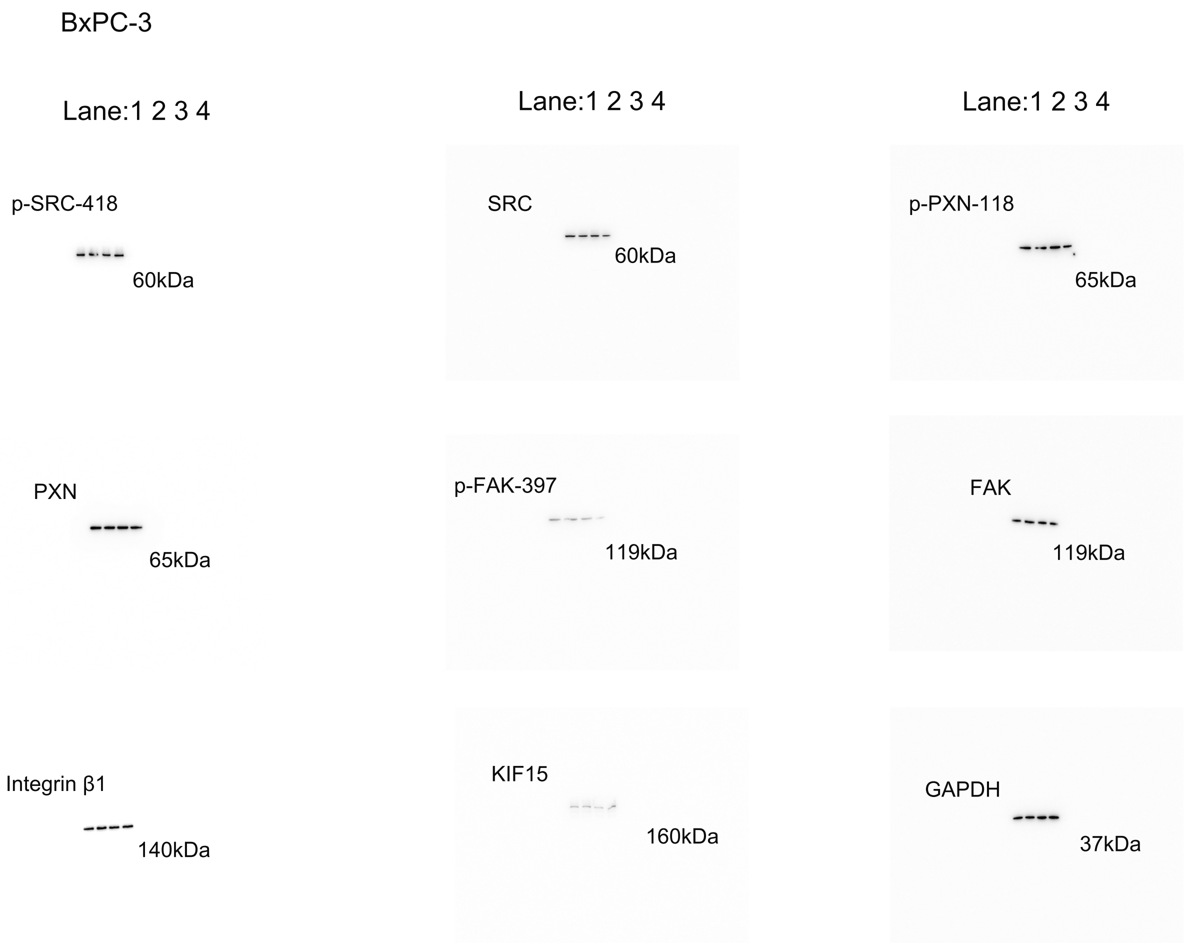
BxPC-3
Lane:1 2 3 4
Lane:1 2 3 4
Lane:1 2 3 4
p-SRC-418
60kDa
SRC
60kDa
p-PXN-118
65kDa
PXN
65kDa
p-FAK-397
119kDa
FAK
119kDa
Integrin β1
140kDa
KIF15
160kDa
GAPDH
37kDa

Supplementary Figure S6C

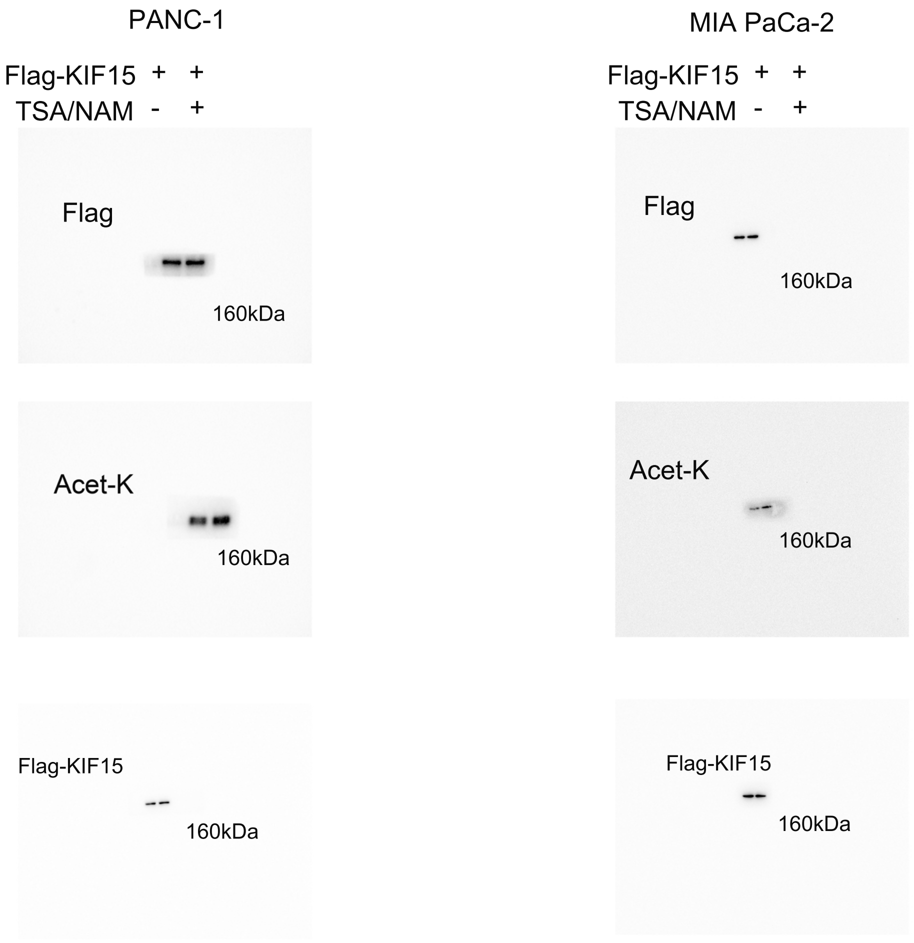

Supplementary Figure S6D

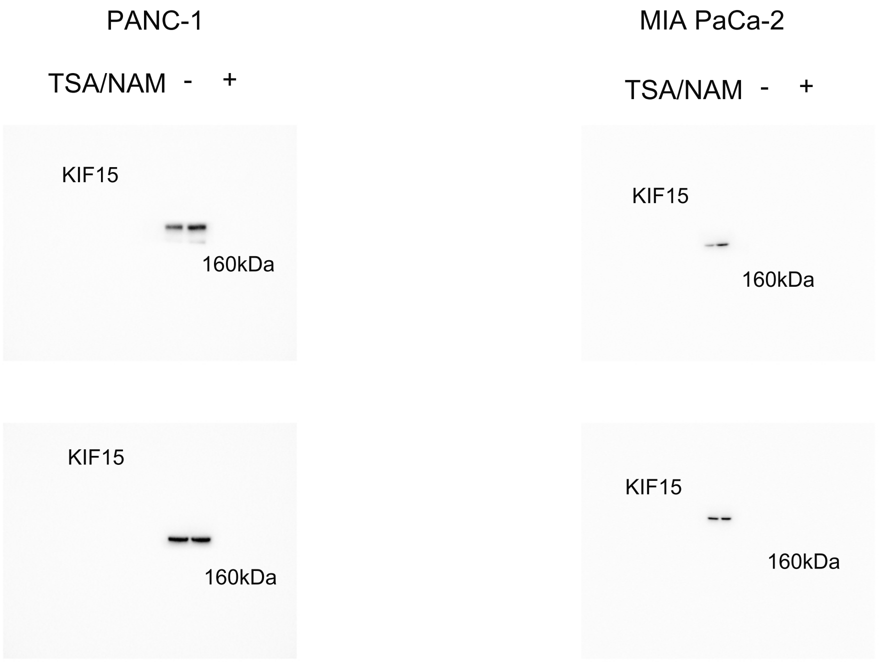

Supplementary Figure S6E

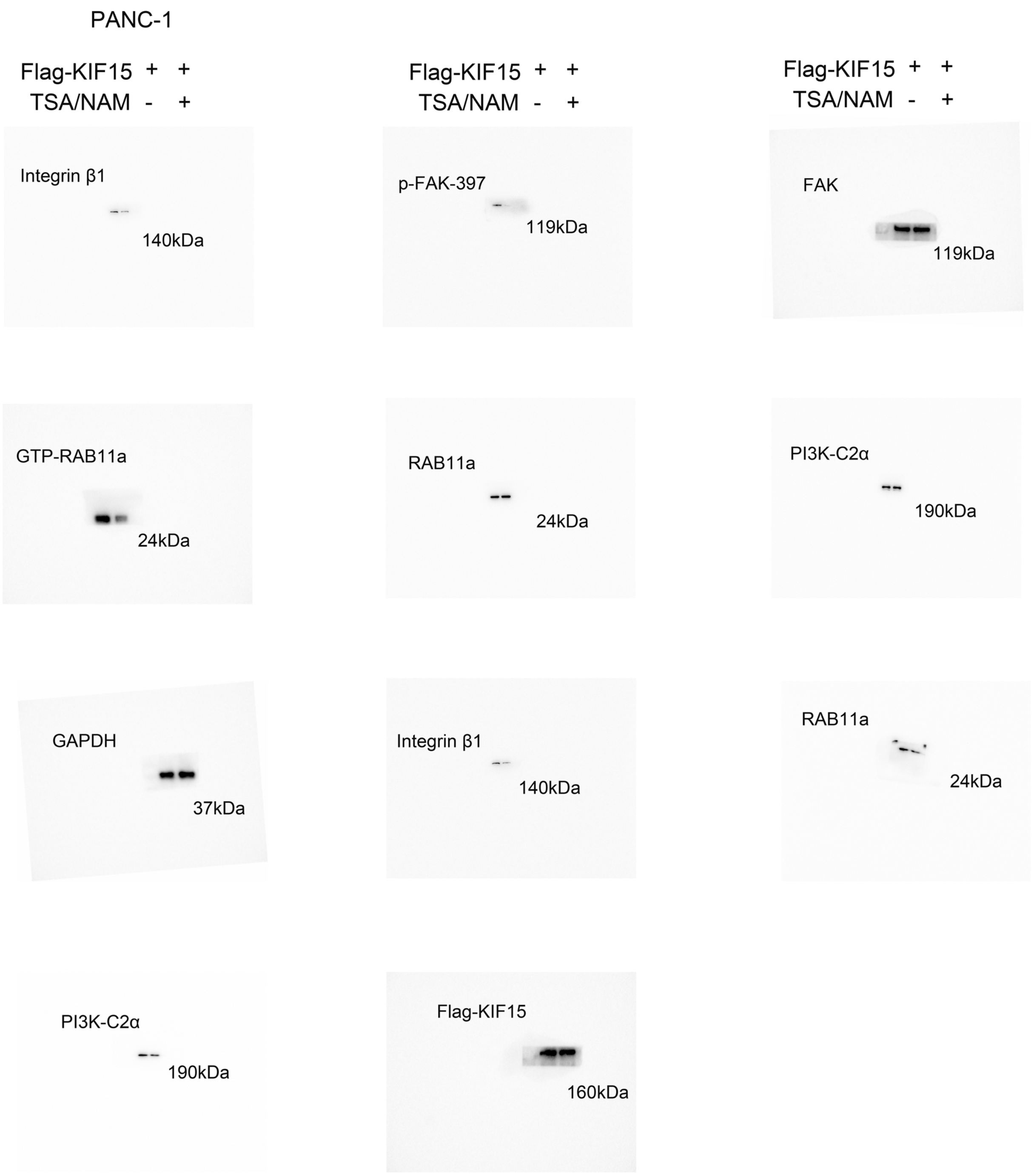

Suppiementary Figure S6E

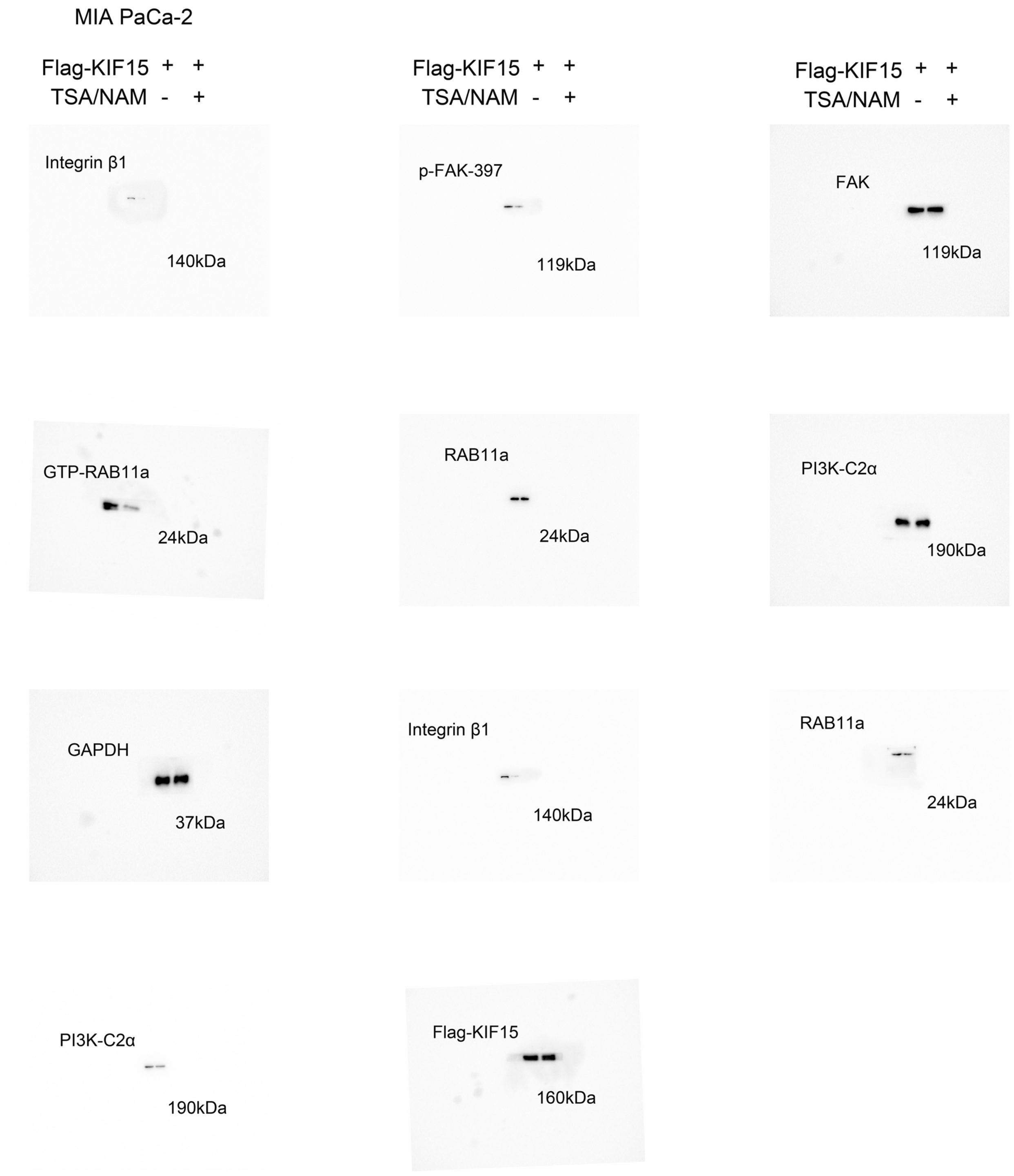

Supplement: Supplementary file 3 — Original Data File [file 41419_2022_5338_MOESM3_ESM.pdf]
